# Effects of exergaming versus endurance training on cardiorespiratory fitness and hemodynamic parameters: a randomized controlled trial

**DOI:** 10.1007/s00421-025-05743-z

**Published:** 2025-03-11

**Authors:** Sascha Ketelhut, Valentin Benzing, Cäcilia Zehnder, Lauren Amor, Yannik Schürch, Manuel Burger, Stefan Schmid, Claudio R. Nigg

**Affiliations:** 1 Institute of Sport Science, University of Bern, Bremgartenstrasse 145, 3013 Bern, Switzerland; 2 Swiss Paraplegic Research, Nottwil, Switzerland; 3 Faculty of Health Sciences and Medicine, University of Lucerne, Lucerne, Switzerland; 4 Ayun, Longevity Clinic, Zurich, Switzerland; 5 School of Health Professions, Bern University of Applied Sciences, Bern, Switzerland; 6 Faculty of Medicine, University of Basel, Basel, Switzerland

**Keywords:** Blood pressure, Heart rate variability, Moderate-intensity continuous exercise, Serious games, VO_2_peak, Enjoyment

## Abstract

**Purpose:**

The study determined whether an exergame training (EXT) resulted in greater improvements in health-related outcomes compared to traditional moderate-intensity continuous training (MICT).

**Methods:**

In total, 47 individuals (age 30±11 years) were randomized into an EXT (n = 24) and an MICT group (n = 23). Throughout the eight-week intervention period, the EXT group attended 20–30 min of EXT three times a week while the MICT group completed 20–45 min of MICT three times a week. Before and after the intervention, BMI, waist-to-height ratio, body fat (BF), resting heart rate (HR), root mean square of successive differences between normal heartbeats (RMSSD), standard deviation of all normal-to-normal intervals (SDNN), average time interval between consecutive R-waves (MeanRR), high-frequency power, low-frequency power, ratio of LF to HF power, enjoyment, systolic (SBP) as well as diastolic blood pressure, and peak oxygen consumption (VO_2_peak) were compared using linear mixed models.

**Results:**

The analyses revealed main effects of time for BF, HR, RMSSD, SDNN, MeanRR, high-frequency power, and SBP (*ps*<.05). A main effect of group was found for enjoyment (*p*<.05) with higher values in the EXT group. Group-by-time interactions (*ps*<.05) were observed for HR, SBP, and VO_2_peak, indicating differential changes over time between groups. The EXT showed a steeper decline in HR and SBP compared to MICT, while demonstrating a greater increase in VO_2_peak.

**Conclusions:**

The EXT was more effective than the MICT in improving VO_2_peak, HR, and SBP. The EXT seems to represent a more effective and more attractive alternative to MICT for health promotion.

**Trial registration number:**

NCT05894031.

**Graphical abstract:**

**Figure:**
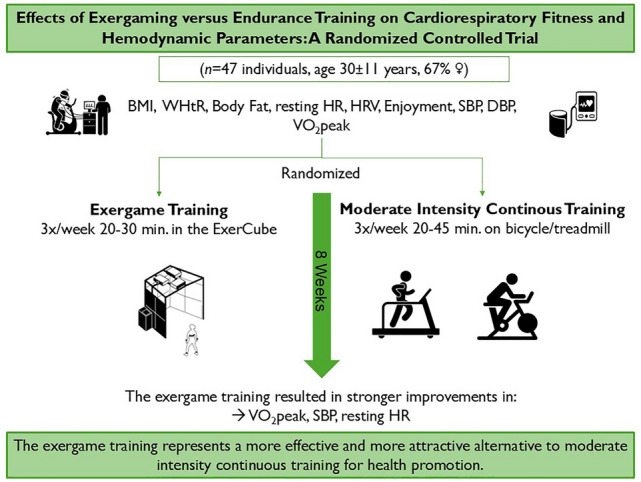
*BMI* Body Mass Index, *WHtR* Waist to height Ratio, *HR* Heart Rate, *HRV* Heart Rate Variability, *SBP* Systolic Blood Pressure, *DBP* Diastolic Blood Pressure, *VO*_2_ *peak* Peak oxygen consumption

**Supplementary Information:**

The online version contains supplementary material available at 10.1007/s00421-025-05743-z.

## Introduction

Engaging in regular physical activity offers numerous health benefits, including a reduced risk of premature mortality and several chronic diseases (Warburton and Bredin 2017). Despite the substantial evidence supporting the positive effects of physical activity, up to 28% of adults worldwide are not physically active enough, representing one of the greatest health-related challenges of our time (Guthold et al. 2018). Several barriers contribute to this inactivity, including lack of time, access to facilities, and motivation (Trost et al. 2002). Enjoyment, or rather the lack thereof, is another critical factor that influences adherence to regular physical activity and exercise routines (Trost et al. 2002). Therefore, it is essential to develop exercise approaches that benefit both, important health-related outcomes and positive affective and enjoyment responses.

Traditionally, exercise guidelines recommend moderate-intensity continuous training (MICT) at an intensity of 60–85% of the maximum heart rate (HRmax) (Piepoli et al. 2014). MICT is widely endorsed due to its confirmed effectiveness in enhancing cardiorespiratory fitness and reducing both cardiovascular and all-cause morbidity and mortality (Parry-Williams and Sharma 2020). Nonetheless, long-term adherence to MICT remains challenging for many people, as it is often perceived as boring or unenjoyable (Trost et al. 2002). In this context, exergaming, which combines physical exercise with interactive video gaming, is discussed as a promising alternative to conventional exercise approaches (Ketelhut et al. 2022b; Röglin et al. 2023a).

Exergaming leverages the engaging and entertaining aspects of video games to promote exercise, potentially overcoming some of the barriers associated with traditional exercise approaches. Previous research has shown that participants report higher enjoyment during exergaming compared to ordinary MICT (Röglin et al. 2021; Ketelhut et al. 2022a). By making exercise more enjoyable, exergaming may encourage more consistent participation, particularly among those who find conventional exercise less appealing. However, despite the promise of exergames' attractiveness, their effectiveness in improving relevant cardiovascular risk factors remains questionable. This is because most exergames only induce relatively low exercise intensity as they are primarily developed for entertainment purposes rather than to promote health benefits (Röglin et al. 2023a). Nonetheless, new exergame concepts are entering the market that aim not only to provide an exciting and engaging gaming experience but also to integrate effective training programs. Findings from this new era of exergames have shown that they are capable of improving key cardiovascular risk factors such as peak oxygen consumption (VO_2_peak) and blood pressure (Berg et al. 2022; Melmer et al. 2024). While these results are promising, empirical evidence is limited because most studies compared exergaming to non-exercise control conditions. While these studies are valuable, they do not clarify how effective exergaming is compared to established exercise approaches, such as MICT. Direct comparisons between exergaming and traditional modalities like MICT are essential to determine its role within the broader landscape of physical activity interventions. Such research provides a clearer understanding of exergaming's relative effectiveness and potential as an alternative for exercise prescription. If exergaming proves to be as effective -or more effective-than MICT in improving health outcomes, while also being more enjoyable and motivating, it could transform physical activity recommendations, particularly for populations that struggle to adhere to traditional exercise programs. However, studies directly comparing exergaming with established exercise programs on key cardiovascular risk factors, such as cardiorespiratory fitness and hemodynamic parameters, remain scarce.

Accordingly, the objective of this study was to compare the effects of an eight-week exergame-training (EXT) program with an eight-week traditional MICT program on cardiorespiratory fitness, anthropometric measurements, and hemodynamic parameters. Based on preliminary studies (Ketelhut et al. 2022c; Kircher et al. 2022a, b), we hypothesized that the EXT program would yield greater improvements in the investigated outcomes compared to the MICT program.

## Methods

### Study design

The study was conducted at the Institute of Sports Science of the University of Bern, using a two-armed, randomized, controlled trial with an intervention period of eight weeks. The study received approval from the Research Ethics Board of the Faculty of Human Sciences at the University of Bern (No. 2021-03-00006) and all participants provided written informed consent. The trial is registered in the ClinicalTrials.gov registry (NCT05894031).

### Participants

The study sample was recruited through word of mouth, social media, mailing lists, and posters placed in public spaces around Bern (Switzerland). After initial contact, potential participants received written information about the study and were screened for eligibility. Participants were eligible if they (1) provided written informed consent, (2) were between 18 and 65 years old, and (3) were not engaged in more than one hour of sports per week. Exclusion criteria included any underlying health conditions or orthopedic injuries that interfere with exercise and regular use of antihypertensive or other cardiovascular medications. Eligible individuals attended the baseline assessment and received randomization assignments via e-mail after the baseline assessment.

The sample size for the study was calculated a priori with G*Power (version 3.1.2; Heinrich Heine University, Dusseldorf, Germany). Based on a medium effect size (*f* = 0.25) for endurance performance (Ketelhut et al. 2022c), with a 5 % level of significance, and with 80 % statistical power, 34 participants were needed. To account for possible dropouts, we included 47 participants.

### Procedure

The participants underwent a baseline assessment, an eight-week intervention period, and a post-intervention assessment. The pre-and post-intervention tests were conducted in the Health Physiology Laboratory. Each participant's pre- and post-tests occurred at the same time of day. All tests were performed by trained study staff using the same equipment under standardized conditions. The temperature of the lab was controlled at 19.4 ± 1.0 °C. Participants were instructed to visit the laboratory at least 4 h postprandial and to refrain from consuming caffeinated or alcoholic beverages and nicotine within four hours prior to testing. Additionally, they were advised to abstain from intensive physical exercise for at least 24 h before the tests. The post-test days were scheduled at least 48 h, but no more than 5 days, after the final intervention session. After the baseline examination, participants were randomized to EXT or the MICT program through block randomization. Allocation was stratified according to VO_2_peak, and the principal investigator carried out the concealed randomization using randomizer.org.

## Measurements

### Anthropometrics

Body height was assessed using a stadiometer. A bioimpedance scale (Tanita RD-545, Tanita Europe BV, Amsterdam, Netherlands) was used to assess body mass and calculate body fat. Waist circumference was measured with a precision of 0.1 cm at the midpoint between the iliac crest and the lowest ribs. Body mass index (BMI) was calculated by dividing a participant’s body mass in kilograms by the square of body height in meters (kg/m^2^). The waist-to-hight ratio (WHtR) was determined by dividing the waist circumference by the body height.

### Physical activity behavior

Self-report physical activity was assessed with the German version of the Godin Leisure Time Exercise Questionnaire (Godin and Shephard 1985; Nigg et al. 2024). This psychometrically robust measure assesses the frequency of mild, moderate, and vigorous exercise and was shown to be valid and reliable (Nigg et al. 2024). From this questionnaire, the weekly minutes of moderate to vigorous physical activity were calculated.

### Cardiac autonomic function

Resting heart rate (HR) and heart rate variability (HRV) were assessed using an HR monitor with a chest strap (Polar H10, Polar Electro OY, Kempele, Finland). After a five-minute period of supine rest to stabilize the HRV signal, a five-minute reading was taken. The RR intervals were recorded at a sampling rate of 1000 Hz. Participants were instructed to breathe normally and refrain from speaking during the measurement. Raw data were processed using the Elite HRV app (Elite HRV Inc, Asheville, United States), which has been evaluated for its validity and reliability (Moya-Ramon et al. 2022). The analysis of HRV included the root mean square of successive differences between normal heartbeats (RMSSD), the standard deviation of all normal-to-normal intervals (SDNN), the average time interval between consecutive R-waves (MeanRR), the high-frequency power (HF-Power), the low-frequency power (LF-Power), and the ratio of LF- to HF-power (LF/HF).

### Blood pressure

Systolic (SBP) and diastolic blood pressure (DBP) measurements were taken using the Mobil-O-Graph (IEM, Stolberg, Germany). This device is clinically validated for hemodynamic measurements (Franssen and Imholz 2010). Custom-fit arm cuffs were placed on the participants' left arms, approximately two centimeters above the antecubital fossa. Following a 10-minute period of supine rest, at least two readings with good data quality were recorded and subsequently averaged for analysis.

### Cardiorespiratory fitness

Participants completed a graded exercise test on a bicycle ergometer (Cyclus2, RBM elektronik-automation GmbH, Leipzig, Germany) to determine their cardiorespiratory fitness level (VO_2_peak). A trained and certified sports scientist continuously supervised the test. The test started at 50 or 75 watts (depending on lean body mass) with a stepwise increment of 25 watts per minute. Participants performed a five-minute warm-up at the respective starting watt level before proceeding to the incremental exercise test. Participants rode on the bicycle until voluntary exhaustion or until a cadence of greater than 60 revolutions per minute could no longer be maintained. During the test, participants were verbally encouraged to reach maximal exhaustion. The test concluded with a three-minute cool-down at 50 watts.

Throughout the test, oxygen consumption was collected and analyzed. Ventilation was recorded continuously using a breath-by-breath gas collection system (Metalyzer 3B, Cortex, Leipzig, Germany). A two-point calibration procedure was conducted according to the manufacturer’s guidelines prior to each testing session. The calibration of the oxygen and carbon dioxide sensors was performed with gases of known concentrations. The flow rate was calibrated with a 2-liter syringe. In addition, ambient air measurements were conducted before each test. The VO_2_peak was defined as the highest recorded value, using the recorded rolling average of 15-second epochs. HR was monitored throughout the test using an HR monitor and a chest strap. The highest HR recorded at the end of the test was defined as HRpeak.

### Enjoyment

Enjoyment was assessed in the second and last week of the intervention period, directly after the respective exercise sessions, using the German version of the Physical Activity Enjoyment Scale (PACES) (Jekauc et al. 2013). This version is widely used in research and has been evaluated as a reliable and valid measurement tool, with internal consistency between 0.92 and 0.93 (Jekauc et al. 2013). Before filling out the questionnaire the participants received standardized instructions on the scale. Participants were asked to rate their current feelings about the completed physical activity on 16 items using a five-point bipolar rating scale. A mean score was calculated for each session.

### Intervention

The EXT consisted of three supervised exergaming sessions per week, which were conducted in the laboratory using the ExerCube device with the game “Sphery Racer” (Sphery AG, Au, Switzerland). The ExerCube is an adaptive fitness game setup that allows the player to engage in a whole-body, functional exercise session. The game is projected onto all three walls surrounding the player, allowing an immersive gaming experience. The player wears HTC Vive trackers on both wrists and both ankles, which continuously track the player’s movements and body position. The objective of the game is to navigate an avatar on a hoverboard through a virtual racetrack by performing various movement tasks. A more detailed explanation of the ExerCube can be found in (Röglin et al. 2021). The gaming sessions lasted between 20 and 30 min, and the duration progressively increased throughout the intervention period (see Supplementary Material 1). Since the game's challenge and speed adjust according to the player's performance, it was not possible to establish a precise exercise intensity. However, previous research has shown that the exercise intensity achieved in the ExerCube is higher than that of a typical MICT (Ketelhut et al. 2022a). Consequently, we chose a shorter exercise duration compared to MICT to help balance the overall workload. Throughout the game, HR was continuously recorded using an HR monitor with a chest strap (Polar Electro Oy, Kempele, Finland).

The MICT comprised three endurance exercise sessions per week. One session per week was supervised by study personnel in the laboratory, utilizing either a bicycle ergometer or a treadmill. The participants conducted the remaining two sessions (jogging) independently, adhering to a structured and individually tailored training plan (see Supplementary Material 1). The exercise duration progressively increased from 20 to 45 minutes. The intensity corresponded to 65 to 75 % HRpeak determined during the incremental exercise test. Participants were equipped with a training watch (Polar Vantage V2, Polar Electro Oy, Kempele, Finland) and an HR monitor with a chest strap to track their exercise sessions.

For both interventions, the Banister training impulse (TRIMP) (Banister 1991) was calculated. Additionally, the mean HR and peak HR for MICT and EXT were calculated by summing the mean HR and peak HR values from each individual exercise session and dividing by the total number of sessions.

### Data analysis

Statistical analyses were carried out using JAMOVI (version 2.3.28.0) and SPSS (version 29). Preliminary analyses were conducted to compare participants’ background characteristics (age, gender, physical activity, body height, body mass, and BMI) between the two groups using independent samples *t*-tests for the continuous variables, and χ^2^-tests for the categorical variables. Training data (mean HR, maximal HR, and TRIMP) were compared using independent samples *t*-tests. Missing data for the PACES were evident due to the study personnel forgetting to distribute the questionnaire. Given the small percentage, missing data were single-imputed using the expectation maximization algorithm. For the t-tests, the assumptions of normality and homogeneity of variances were evaluated. Normality was assessed using Shapiro-Wilk tests and visual inspection of histograms and Q-Q plots. Homogeneity of variances was tested using Levene’s test. No violations of these assumptions were detected.

For the main analyses, separate linear mixed models were employed to analyze the effects of group, time, and their interaction (group*time) on the dependent variables using the GAMLj package. The model included the participant as a random effect to account for the clustering within participants. Body mass was included as a covariate to control for its potential confounding effect because weight differed significantly between the two groups at the pre-test. In case of significant time effects, simple effects analyses using time as a simple effects variable and group as moderator were conducted. To ensure the validity of the models used in our analysis, we systematically evaluated the following assumptions: Linearity between predictors and the outcome variable was confirmed through visual inspection of scatterplots and partial residual plots. The randomness and normality of residuals were assessed using quantile-quantile (Q-Q) plots and histograms, supported by the Shapiro-Wilk test where appropriate. Homoscedasticity was evaluated by inspecting plots of residuals against fitted values to confirm the absence of patterns or variance trends. The normality of residual errors was further validated through Q-Q plots. Overall, these diagnostic evaluations indicated that the assumptions of the model were met.

## Results

**Fig. 1:**
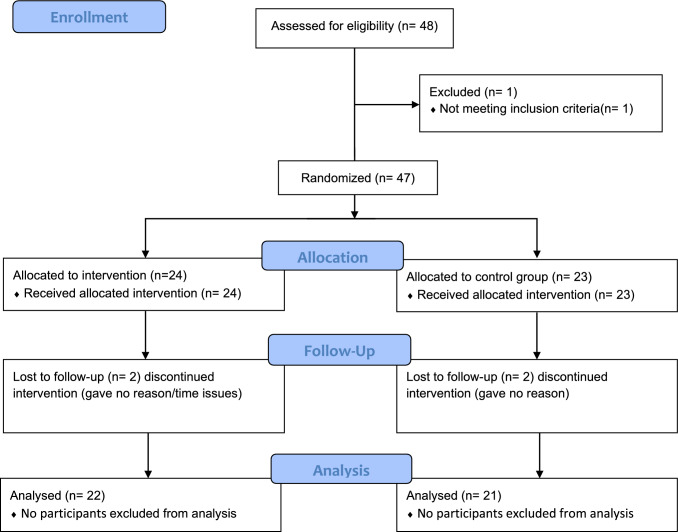
CONSORT Flow-Diagram

Four participants (EXT = 2) dropped out of the study due to time constraints (see Flow-chart Fig. 1), leaving a total of 43 participants (30 ± 11 years, 29 female) for the final analyses. No adverse events were reported among the participants during the intervention period. There were no differences between the two groups at baseline except for body mass, which was higher in the MICT group compared to the EXT group (Table 1). According to their minutes of moderate to vigorous physical activity, 30 participants (EXT = 16) did not meet the World Health Organization's physical activity recommendations of >150 minutes per week and could therefore be classified as physically inactive (World Health Organization 2019). Regarding BMI, 10 participants (EXT = 5) were classified as overweight, and three participants (EXT = 1) were classified as obese. In terms of WHtR, nine participants (EXT = 3) were above the established health risk threshold of 0.5 for WHtR (Ashwell et al. 2012). Based on VO_2_peak cutoff values (Kaminsky et al. 2017), participants were classified into the following categories: five participants (EXT = 3) as superior, 13 participants (EXT = 5) as excellent, 13 participants (EXT = 9) as good, seven participants (EXT = 2) as fair, and five participants (EXT = 3) as poor. Four participants (EXT = 4) had high-normal blood pressure, and four participants (EXT = 1) were classified as hypertensive.

**Table 1:** Comparison of participants’ characteristics for exergame training (EXT) group and moderate-intensity continuous training (MICT) group

	EXT		MICT				95% CI	
	*M*	*SD*	*M*	*SD*	*p*	*d*	Lower	Upper
Age (years)	30	12	29	10	.738	0.1	−0.5	0.7
MVPA (min.)	127	121	157	175	.516	−0.20	−0.8	0.4
Body Height (m)	1.7	0.06	1.74	0.09	.135	−0.46	−1.07	0.15
Body Mass (kg)	66.8	10.8	75.7	11	.01	−0.82	−1.46	−0.17
BMI (kg/m^2^)	23.1	3.4	25.2	3.6	.056	−0.6	−1.22	0.03
WHtR	0.44	0.05	0.46	0.07	.351	−0.39	−0.89	0.32

*M* = Mean, *SD* = Standard deviation, *CI* = Confidence interval, *MVPA* = Moderate to vigorous physical activity, *BMI* = Body-mass-index, *WHtR* = Waist-to-height ratio

The analyses of training data revealed that exercise intensity was notably higher during the EXT program, as evidenced by significantly higher mean HR (158 ± 10 min^−1^ vs. 137 ± 9 min^−1^, *p* <.001), peak HR (188 ± 12 min^−1^ vs. 151 ± 10 min^−1^, *p* <.001), and percentage of HRpeak (84.7 ± 5.3 % vs. 73.9 ± 3.3 %, *p* <.001), in comparison to the MICT program. Conversely, the TRIMP was greater in the MICT group (1684 ± 392 vs. 2822 ± 584, *p* <.001). In terms of compliance, participants in the EXT group attended a higher number of sessions than those in the MICT group (23.4 ± 1.4 days vs. 21.5 ± 3.1 days, *p* = .013).

The main analyses and descriptive statistics are presented in Tables 2 and 3 and Supplementary Material 2, while a comprehensive overview of the statistical models is provided in Supplementary Material 3. The outcome analysis revealed a significant main effect of time on body fat percentage (*F*(1, 39.90) = 14.12, *p* < .001, 95% CI [−1.86, −0.59]), showing a reduction in the MICT group (*p* = .001), but not in the EXT group (*p* = .079). In terms of cardiac autonomic function, significant main effects of time were observed for resting HR (*F*(1, 41.12) = 19.22, *p* < .001, 95% CI [−6.01, −2.30]), with a reduction in the EXT group (*p* < .001) but not in the MICT group (*p* = .195). Similarly, RMSSD (*F*(1, 41.23) = 16.27, *p* < .001, 95% CI [5.86, 16.95]) increased in the EXT group (*p* < .001), but not in the MICT group (*p* = .154). A significant main effect of time for SDNN (*F*(1, 41.23) = 21.25, *p* < .001, 95% CI [7.45, 18.48]) was found, with increases in both the EXT (*p* = .006) and MICT (*p* < .001) groups. Furthermore, a significant effect of time was found for MeanRR (*F*(1, 41.20) = 17.69, *p* < .001, 95% CI [28.35, 77.83]), with an increase in the EXT group (*p* < .001), but not in the MICT group (*p* = .097). HF-Power also showed a significant time effect (*F*(1, 41.42) = 10.80, *p* = .002, 95% CI [238.59, 943.94]), with an increase in the EXT group (*p* = .004), but not in the MICT group (*p* = .117). For hemodynamic parameters, significant time effects were found for SBP (*F*(1, 41.30) = 30.81, *p* < .001, 95% CI [−6.30, −3.01]), with reductions in both the EXT (*p* < .001) and MICT (*p* = .048) groups, and for DBP (*F*(1, 41.29) = 4.53, *p* = .039, 95% CI [−3.42, −0.14]), with a reduction in the EXT group (*p* = .010), but not in the MICT group (p = .734). Additionally, a significant time effect was observed for VO_2_peak (*F*(1, 41.35) = 40.24, *p* < .001, 95% CI [1.55, 2.94]), with an increase in the EXT group (*p* < .001), but not in the MICT group (*p* = .143). A significant main effect of group was found for physical activity enjoyment (*F*(1, 40.48) = 5.41, *p* = .025, 95% CI [−0.95, 40.48]), with higher enjoyment reported in the EXT condition compared to the MICT condition.

**Table 2:** Descriptive statistics for outcome variables for exergame training group (EXT) and moderate-intensity continuous training group (MICT) before (pre) and after (post) the intervention

		Pre		Post	
Outcome	Group	*M*	*SD*	*M*	*SD*
BF (%)	EXT	28.2	9.3	27.2	8.5
	MICT	30	7.4	28.2	6.9
Resting HR (min^−1^)	EXT	69	7	63	7
	MICT	67	9	66	9
RMSSD (ms)	EXT	47.89	16.41	65.17	23.33
	MICT	58.75	35.38	65.01	37.35
SDNN (ms)	EXT	61.17	20.02	72.92	24.71
	MICT	64.96	30.63	79.81	33.04
MeanRR (ms)	EXT	881.44	85.51	957.1	92.05
	MICT	915.57	127.67	946.31	155.75
LF/HF	EXT	2.1	2.31	1.41	1.1
	MICT	1.28	0.97	1.32	1.34
LF-Power (ms^2^)	EXT	1718.37	1670.67	1888.87	1823.57
	MICT	1565.68	1720.83	2433.01	3101.87
HF-Power (ms^2^)	EXT	998.63	671.03	1796.92	1506.65
	MICT	2208.87	3578.8	2652.23	3449.63
SBP (mmHg)	EXT	120	9	113	8
	MICT	119	11	116	11
DBP (mmHg)	EXT	74	9	71	9
	MICT	74	9	73	8
VO_2_peak (mL/kg/min)	EXT	35.21	10.06	38.96	10.52
	MICT	35.85	7.1	36.62	7.46
PACES	EXT	68.8	5.9	68.9	6.8
	MICT	62.9	11.7	62.8	9.1

*M* = Mean, *SD* = Standard deviation, *BF* = Body fat percentage, *resting HR* = Resting heart rate, *RMSSD* = Root mean square of successive differences between normal heartbeats, *SDNN* = Standard deviation of all normal-to-normal intervals, *MeanRR* = Average time interval between consecutive R-waves, *HF-Power* = High-frequency power, *LF-Power* = Low-frequency power, *LF/HF* = Ratio of LF- to HF-power, *SBP* = Systolic blood pressure, *DBP* = diastolic blood pressure, *VO*_*2*_*peak* = Peak oxygen consumption, *PACES* = Physical activity enjoyment score

**Table 3:** Fixed effects parameter estimates for the different outcome variables

				95% CI				
	Effect	Estimate	SE	Lower	Upper	*df*	*F*	*p*
BMI (kg/m^2^)	Intercept	23.99	0.34	23.33	24.65	39.21	71.6	< .001
	Group	−0.91	0.68	−2.24	0.42	41.12	−1.34	.188
	Time	−0.03	0.03	−0.08	0.02	39.11	−1.06	.294
	Body Mass	0.34	0.01	0.31	0.36	53.41	26.66	< .001
	Group × Time	−0.02	0.05	−0.13	0.08	38.53	−0.45	.652
WHtR	Intercept	0.45	0.01	0.44	0.46	40.04	79.27	< .001
	Group	−0.01	0.01	−0.03	0.02	40.34	−0.42	.678
	Time	0	0.01	−0.01	0.01	41.11	0.31	.756
	Body Mass	0	0	0	0	42.09	5.69	< .001
	Group × Time	0.01	0.01	−0.02	0.03	41	0.77	.445
BF (%)	Intercept	28.37	1.18	26.05	30.69	38.54	24.01	< .001
	Group	−1.92	2.5	−6.83	2.99	41.25	−0.77	.447
	Time	−1.22	0.33	−1.86	−0.59	39.9	−3.76	< .001
	Body Mass	0.38	0.09	0.19	0.56	68.46	4.04	< .001
	Group × Time	−0.81	0.65	−2.08	0.45	38.95	−1.26	.215
Mean HR (min^−1^)	Intercept	66.33	1.11	64.15	68.5	40.02	59.73	< .001
	Group	−0.03	2.4	−4.74	4.67	40.5	−0.01	.989
	Time	−4.16	0.95	−6.01	−2.3	41.12	−4.38	< .001
	Body Mass	0.05	0.1	−0.15	0.25	43.5	0.46	.647
	Group × Time	4.74	1.89	1.03	8.45	40.94	2.5	.016
RMSSD (ms)	Intercept	59.29	4.08	51.3	67.28	40.08	14.55	< .001
	Group	12.66	8.79	−4.58	29.89	40.79	1.44	.158
	Time	11.4	2.83	5.86	16.95	41.23	4.03	< .001
	Body Mass	−0.82	0.37	−1.55	−0.09	45.35	−2.21	.032
	Group × Time	−11.07	5.64	−22.14	−0.01	40.96	−1.96	.057
SDNN (ms)	Intercept	69.79	3.8	62.34	77.25	40.1	18.34	< .001
	Group	11.96	8.22	−4.14	28.06	40.73	1.46	.153
	Time	12.96	2.81	7.45	18.48	41.23	4.61	< .001
	Body Mass	−0.74	0.35	−1.43	−0.06	44.74	−2.14	.038
	Group × Time	3.03	5.62	−7.97	14.04	40.99	0.54	.592
MeanRR (ms)	Intercept	925.13	17.07	891.67	958.59	40.07	54.19	< .001
	Group	13.77	36.86	−58.47	86.02	40.7	0.37	.711
	Time	53.09	12.62	28.35	77.83	41.2	4.21	< .001
	Body Mass	−0.24	1.56	−3.3	2.82	44.71	−0.15	.881
	Group × Time	−44.93	25.21	−94.33	4.48	40.96	−1.78	.082
LF/HF	Intercept	1.53	0.21	1.11	1.94	40.05	7.23	< .001
	Group	−0.42	0.46	−1.31	0.48	40.41	−0.92	.365
	Time	−0.32	0.21	−0.73	0.09	41.13	−1.54	.131
	Body Mass	0	0.02	−0.04	0.03	42.63	−0.23	.821
	Group × Time	0.72	0.42	−0.1	1.54	40.99	1.73	.092
LF-Power (ms^2^)	Intercept	1907.79	289.7	1339.99	2475.59	40	6.59	< .001
	Group	737.87	626.36	−489.78	1965.52	40.43	1.18	.246
	Time	491.5	261.66	−21.35	1004.36	41.09	1.88	.067
	Body Mass	−60.89	26.74	−113.29	−8.49	43.11	−2.28	.028
	Group × Time	692.5	522.78	−332.13	1717.12	40.92	1.32	.193
HF-Power (ms^2^)	Intercept	1920.96	370.58	1194.65	2647.28	40.17	5.18	< .001
	Group	1617.48	796.31	56.74	3178.21	41.47	2.03	.049
	Time	591.27	179.94	238.59	943.94	41.42	3.29	.002
	Body Mass	−65.67	32.72	−129.79	−1.55	50.84	−2.01	.05
	Group × Time	−359.59	358.68	−1062.59	343.41	40.94	−1	.322
SBP (mmHg)	Intercept	117.07	1.35	114.43	119.71	40.13	86.78	< .001
	Group	−1.5	2.91	−7.2	4.2	40.98	−0.51	.61
	Time	−4.65	0.84	−6.3	−3.01	41.3	−5.55	< .001
	Body Mass	0.32	0.12	0.08	0.56	46.68	2.61	.012
	Group × Time	4.43	1.67	1.15	7.71	40.97	2.65	.012
DBP (mmHg)	Intercept	73.06	1.22	70.67	75.44	40.12	60.03	< .001
	Group	−1.41	2.63	−6.56	3.74	40.84	−0.54	.595
	Time	−1.78	0.84	−3.42	−0.14	41.27	−2.13	.039
	Body Mass	0.24	0.11	0.02	0.45	45.49	2.13	.039
	Group × Time	2.74	1.67	−0.53	6.02	41	1.64	.108
HRmax (min^−1^)	Intercept	185.42	1.66	182.17	188.67	40.15	111.78	< .001
	Group	−0.46	3.57	−7.47	6.54	41.1	−0.13	.898
	Time	−0.94	0.97	−2.84	0.96	41.34	−0.97	.336
	Body Mass	0.01	0.15	−0.28	0.3	47.56	0.06	.951
	Group × Time	1.11	1.93	−2.68	4.9	40.98	0.57	.57
VO_2_peak (mL/kg/min)	Intercept	36.66	1.36	33.99	39.33	40	26.92	< .001
	Group	−0.67	2.88	−6.32	4.97	42.86	−0.23	.816
	Time	2.25	0.35	1.55	2.94	41.35	6.34	< .001
	Body Mass	−0.02	0.11	−0.23	0.19	71.62	−0.19	.848
	Group × Time	−2.99	0.7	−4.36	−1.61	40.36	−4.26	< .001
MVPA (min)	Intercept	145.99	18.23	110.26	181.71	40.05	8.01	< .001
	Group	17.34	39.43	−59.94	94.61	40.41	0.44	.662
	Time	8.67	18.12	−26.84	44.18	41.13	0.48	.635
	Body Mass	0.1	1.69	−3.21	3.41	42.62	0.06	.952
	Group × Time	−23.43	36.2	−94.38	47.52	40.99	−0.65	.521
PACES	Intercept	65.86	1.21	63.5	68.22	40.07	54.62	< .001
	Group	−6.06	2.61	−11.17	−0.95	40.48	−2.32	.025
	Time	−0.04	1.13	−2.25	2.17	41.15	−0.04	.972
	Body Mass	0.01	0.11	−0.21	0.22	42.97	0.05	.957
	Group × Time	−0.26	2.25	−4.67	4.15	41	−0.11	.909

*SE* = Standard error, *CI* = Confidence interval, *BMI* = Body Mass Index, *WHtR* = Waist-to-height-ratio, *BF* = Body fat, *resting HR* = Resting heart rate, *RMSSD* = Root mean square of successive differences between normal heartbeats, *SDNN* = Standard deviation of all normal-to-normal intervals, *MeanRR* = Average time interval between consecutive R-waves, *HF-Power* = High-frequency power, *LF-Power* = Low-frequency power, *LF/HF* = Ratio of LF- to HF-power, *SBP* = Systolic blood pressure, *DBP* = Diastolic blood pressure, *HRmax* = Heart rate max, *VO*_*2*_*peak* = Peak oxygen consumption, *MVPA* = Minutes of moderate to vigorous physical activity, *PACES* = Physical activity enjoyment

**Fig. 2:**
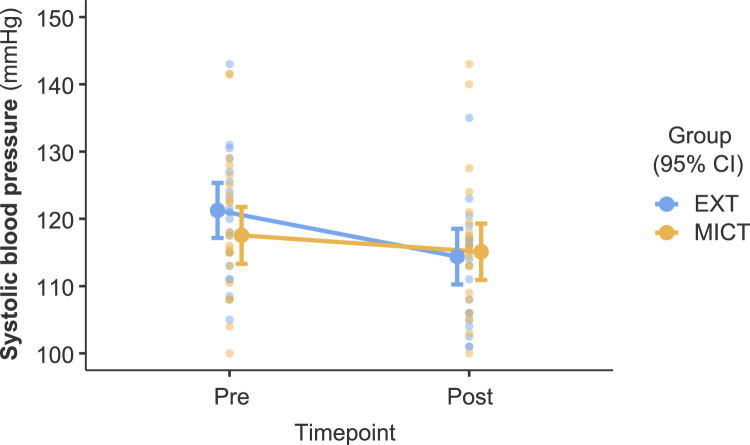
Estimated marginal means of the systolic blood pressure comparing the exergame training group (EXT) and the moderate-intensity continuous training group (MICT). Error bars represent 95% CI

**Fig. 3:**
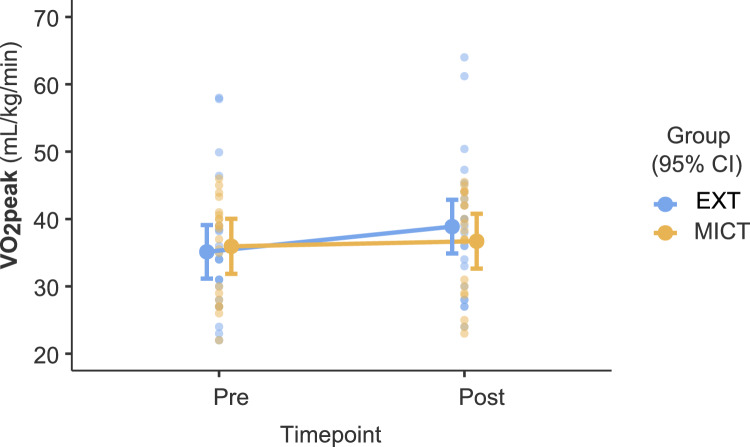
Estimated marginal means of the VO_2_peak comparing the exergame training group (EXT) and the moderate-intensity continuous training group (MICT). Error bars represent 95% CI

Notably, significant group*time interactions were observed for resting HR (*F*(1, 40.94) = 6.26, *p* = .016, 95% CI [1.03, 8.45]), SBP (*F*(1, 40.97) = 7.00, *p* = .012, 95% CI [1.15, 7.71]) (see Fig. 2), and VO_2_peak (*F*(1, 40.36) = 18.11, *p* < .001, 95% CI [−4.36, −1.61]) (see Fig. 3), indicating differential changes over time between groups. The decrease in resting HR and SBP was steeper in the EXT condition than MICT, whereas the increase in VO_2_peak was higher in the EXT condition.

## Discussion

The present study compared the effects of an eight-week EXT program with a traditional MICT program on cardiorespiratory fitness, anthropometric measurements, and hemodynamic parameters. Overall, both exercise programs resulted in improvements in body fat percentage, VO_2_peak, blood pressure, and cardiac autonomic function. However, the EXT intervention led to more pronounced enhancements in VO_2_peak, SBP, and resting HR. Moreover, participants reported greater enjoyment during the EXT compared to the MICT.

Body fat, particularly its unfavorable distribution in the abdominal area, contributes to an increased risk of cardiovascular disease (Powell-Wiley et al. 2021). In agreement with current research (Wewege et al. 2017), we found a reduction in body fat percentage after the exercise interventions. However, no interaction effects were observed in any of the anthropometric parameters. This is surprising, as we had anticipated a stronger effect on anthropometric parameters for the EXT group due to the greater muscle mass recruitment during the functional exercise sessions. A recent umbrella review on the impact of exergaming on body composition, BMI, and other weight-related outcomes reveals inconsistent results (O’Loughlin et al. 2020). Research indicates that the effectiveness of EXT on anthropometric parameters is more robust in studies involving higher exercise frequency (more than three times per week) and in more obese individuals (O’Loughlin et al. 2020). Therefore, the predominantly normal-weight sample in this study and the lower exercise frequency may have impacted the results. Additionally, most previous studies focus on general improvements through regular exergaming rather than direct comparisons with other exercise programs, such as MICT.

Consistent with our hypothesis, the EXT intervention led to a more pronounced increase in VO_2_peak compared to MICT. While the positive effects of MICT on VO_2_peak are well-documented (Milanović et al. 2015), the effects of EXT are less frequently studied and yield inconclusive results. Studies by Berg et al. (2022), and Melmer et al. (2024) have shown significant improvements in VO_2_peak after six and eight weeks of EXT, respectively, although these studies did not include active comparison groups. Warburton et al. (2007) reported greater VO_2_peak improvements with EXT compared to MICT; however, the study’s small sample size and unstructured MICT regimen limit the strength of its conclusions. In contrast, Jo et al. (2020) found that both EXT and structured MICT led to significant improvements in VO_2_peak in postmenopausal women with high cardiovascular risk, with no significant difference between the two interventions. Taken together, the current study adds to the empirical evidence indicating that EXT benefits VO_2_peak compared to MICT. This finding bears significant relevance, given that VO_2_peak stands out as a robust predictor of both mortality and morbidity, outweighing other traditional risk factors (Imboden et al. 2018). The observed increase in VO_2_peak of 3.74 mL/kg/min in the EXT group is clinically significant, as each 1-mL/min/kg increase in VO_2_peak is associated with an approximately 11 % decrease in mortality risk (Imboden et al. 2019).

Both interventions led to a reduction in blood pressure. However, the EXT group exhibited a significantly greater reduction in SBP compared to the MICT group. The absolute reduction in SBP of 7 mmHg observed in the EXT group exceeds the 4 mmHg reduction reported for MICT in a recently published systematic review of meta-analyses (Hanssen et al. 2022). In contrast, the MICT group in our study experienced a reduction of 3 mmHg, which is slightly lower. Notably, the meta-analyses included individuals across all blood pressure categories, while our study primarily involved normotensive participants. When considering only individuals with normal blood pressure, the meta-analyses reported a mean reduction of 2 mmHg for MICT (Hanssen et al. 2022). Only two prior studies have compared EXT to alternative exercise programs. Warburton et al. (2007) observed a significantly greater reduction in SBP within the EXT group compared to a comparison group engaged in an unstructured exercise program. In contrast, Jo et al. (2020) found no differences in blood pressure between an EXT program and a MICT program in high-risk patients.

After the intervention period, we observed overall reductions in resting HR and increases in RMSSD, SDNN, MeanRR, and HF-Power. As expected, the reduction in resting HR was significantly more pronounced in the EXT group. Additionally, there was a statistical tendency toward interactions for RMSSD, SDNN, MeanRR, and LF/HF, favoring the EXT condition.

The reduction in resting HR is in line with Melmer et al. (2024) who assessed the effects of a very similar EXT and compared it to an inactive control group. The association between elevated HR and higher cardiovascular disease incidence and mortality (Vazir et al. 2018) underscores the importance of our findings. The reduction of 6.5 min^−1^ in the present study is relevant, considering that an increase in HR by 5 min^−1^ has been associated with a 12 % higher risk of all-cause mortality (Vazir et al. 2018).

While it is widely accepted that regular MICT increases parasympathetic activity (Grässler et al. 2021), studies assessing the effects of EXT on HRV parameters are sparse. In a previous study, Villafaina et al. (2020) found a significantly stronger reduction in SDNN after a 24-week EXT intervention compared to an inactive control group in women with fibromyalgia. Unfortunately, studies comparing the effects of EXT to other exercise approaches are still lacking. The positive effects on HRV are relevant, given that HRV is recognized as an independent risk factor for morbidity and mortality and is an early marker of cardiovascular risk (Thayer et al. 2010).

The more pronounced effects on VO_2_peak and hemodynamic parameters detected in the EXT group compared to the MICT group in our study could be attributed to the higher exercise intensity attained in the EXT, despite a markedly lower exercise volume and total workload (1684 vs. 2822 TRIMP). The mean exercise intensity during the EXT reached 84.7 ± 5.2 % of HRpeak, while the mean exercise intensity in the MICT group reached only 73.9 ± 3.3 % of HRpeak. Previous studies have demonstrated that higher exercise intensity results in greater improvements in VO_2_peak (Milanović et al. 2015), attributed to stronger enhancements in central oxygen delivery through increased stroke volume and cardiac output (Lepretre et al. 2004). Higher exercise intensities are also associated with more beneficial effects on systolic blood pressure (SBP) and cardiac autonomic function (Grässler et al. 2021). These benefits are attributed to greater reductions in oxidative stress and sympathetic nervous activity, as well as enhanced distribution and production of shear stress-induced nitric oxide (Pattyn et al. 2015). The high exercise intensity attained in the ExerCube in the present study may also explain the more pronounced effects observed compared to previous research on EXT. While not all exergame studies report the exercise intensity attained during the intervention, the intensity during the EXT in the present study was higher compared to that in the study by Berg et al. (2022) (mean HR 73.7%) and Jo et al. (2020) (60–80% of HR reserve). The relatively high exercise intensity during the EXT can be attributed to the game setup and innovative game design, as discussed previously (Ketelhut et al. 2022b).

Apart from the health-related outcomes the participants in the EXT group reported significantly higher enjoyment during the selected EXT sessions compared to the selected MICT sessions in the comparison group. This finding is significant, as research suggests that perceived enjoyment during exercise is linked to higher levels of participation (Dunton and Vaughan 2008). Our findings align with previous studies demonstrating that exergaming elicits more positive emotional responses and greater enjoyment compared to traditional exercise methods (Bock et al. 2021; Röglin et al. 2021, 2023b). Notably, our study revealed that enjoyment scores were higher for the EXT group, despite the significantly higher exercise intensity. Previous research indicates that higher exercise intensities lead to a decline in pleasure (Ekkekakis et al. 2011). Therefore, our results support the use of the ExerCube device as an attractive tool for promoting high-intensity physical activity. The greater perceived enjoyment during the EXT may be attributed to the immersive and engaging experience provided by the audio-visual game scenario (Röglin et al. 2021). This experience likely redirects attentional focus, helping to dissociate from the unpleasant sensations induced by exertion, thereby reducing negative feelings during exercise (Chow and Etnier 2017). Importantly, enjoyment levels remained consistently high throughout the eight-week intervention period. Nevertheless, it remains uncertain whether this effect would endure over longer intervention periods.

## Limitations

There are methodological limitations that warrant consideration. First, the results are specific to the game setting (ExerCube) and the game scenario (Sphery Racer) used in this study. Different exergames are likely to produce varying effects. Second, the EXT and the MICT were not workload-matched as it was difficult to anticipate the exercise intensity in the ExerCube, due to the fact that it is adjusted according to the in-game performance. Third, only one of the three exercise sessions in the MICT group was supervised, while the other two were performed independently. Although exercise sessions were tracked using a sports watch to ensure participants completed the prescribed workouts at the required intensity, this limited supervision may have contributed to the lower compliance observed in the MICT group. Fourth, body fat percentage was assessed using a bioimpedance scale, which is not considered the gold standard for determining body composition. Lastly, the study included a relatively small sample size and focused exclusively on healthy individuals. Therefore, the results cannot be generalized to other populations. Future studies with larger sample sizes and more diverse populations, particularly high-risk patients, are needed to better understand the potential of this approach in different contexts.

## Conclusion

In conclusion, the eight-week EXT program resulted in stronger improvements in VO_2_peak, hemodynamic parameters, and cardiac autonomic function compared to the MICT program. Additionally, participants in the EXT group reported consistently higher levels of enjoyment throughout the intervention period. These results suggest that the ExerCube is not only a more effective and time-efficient alternative to traditional MICT but also a more appealing option for health promotion. Future investigations should explore whether these outcomes remain consistent across diverse target demographics and when compared against other established exercise modalities. Longer intervention periods should also be considered to evaluate their sustained impact on enjoyment. Furthermore, conducting a more comprehensive analysis of various exergames is essential to clarify which specific games and exergame devices are most effective.

## Supplementary Information

Below is the link to the electronic supplementary material.

Supplementary file1 (DOCX 18 KB)

Supplementary file2 (DOCX 33 KB)

Supplementary file3 (DOCX 670 KB)

Supplementary file4 (DOC 219 KB)

## Data Availability

All raw data are available on request to the corresponding author.

## Abbreviations

BMI: Body mass index
DBP: Diastolic blood pressure
EXT: Exergame-training
HF-Power: High-frequency power
HR: Heart rate
HRmax: Maximum heart rate
HRV: Heart rate variability
LF/HF: Ratio of low-frequency power to high-frequency power
LF-Power: Low-frequency power
MICT: Moderate-intensity continuous training
MeanRR: Average time interval between consecutive R-waves
PACES: Physical Activity Enjoyment Scale
RMSSD: Root mean square of successive differences between normal heartbeats
SBP: Systolic blood pressure
SDNN: Standard deviation of all normal-to-normal intervals
TRIMP: Training impulse
VO2peak: Peak oxygen consumption
WHtR: Waist-to-hight ratio
