# Supplementary material for: Effects of exergaming versus endurance training on cardiorespiratory fitness and hemodynamic parameters: a randomized controlled trial
Source: Eur J Appl Physiol. 2025 Mar 11;125(7):1817–30. doi: 10.1007/s00421-025-05743-z (PMC12227349; doi:10.1007/s00421-025-05743-z)
Supplement: Supplementary file 1 — Supplementary file1 (DOCX 18 KB) [file 421_2025_5743_MOESM1_ESM.docx]

Supplemental Digital Content 1: Exemplary Training Protocol for exergame training (EXT) and the moderate-intensity continuous training (MICT)

| **Training session** | **EXT** | **MICT** |
| --- | --- | --- |
| 1 | Sphery Racer (2 x 10 min. with 5 min. rest between games) | Jogging (10 + 10 min. at 65-70 % HRmax, 2 min. passive rest) |
| 2 | Sphery Racer (2 x 10 min. with 5 min. rest between games) | Jogging (20 min. at 65-70 % HRmax) |
| 3 | Sphery Racer (1 x 10 min. + 1 x 15 min. with 5 min. rest between games) | Bicycle riding (20 min. at 65-70 % HRmax) |
| 4 | Sphery Racer (1 x 10 min. + 1 x 15 min. with 5 min. rest between games) | Jogging (20 min. at 65-70 % HRmax) |
| 5 | Sphery Racer (1 x 10 min. + 1 x 15 min. with 5 min. rest between games) | 20 min. jogging (65-70 % HRmax) |
| 6 | Sphery Racer (2 x 15 min. with 5 min rest between games) | Bicycle riding (25 min. at 65-70 % HRmax) |
| 7 | Sphery Racer (20 min.) | Jogging (15 + 10 min. at 65-75 % HRmax, 2-min. walking break) |
| 8 | Sphery Racer (20 min.) | Jogging (20 + 10 min at 65-75 % HRmax, 2-min. walking break) |
| 9 | Sphery Racer (20 min.) | Bicycle riding (25 min. at 65-70 % HRmax) |
| 10 | Sphery Racer (25 min.) | Jogging (25 min. at 65-70 % HRmax) |
| 11 | Sphery Racer (25 min.) | Jogging (30 min. at 65-75 % HRmax) |
| 12 | Sphery Racer (25 min.) | Bicycle riding (30 min. at 65-70 % HRmax) |
| 13 | Sphery Racer (1 x 20 min. + 1 x 10 min. with 5 min rest between games) | Jogging (35 min. at 65-70 % HRmax) |
| 14 | Sphery Racer (30 min.) | Jogging (35 min. at 65-75 % HRmax) |
| 15 | Sphery Racer (30 min.) | Bicycle riding (35 min. at 65-70 % HRmax) |
| 16 | Sphery Racer (30 min.) | Jogging (35 min. at 65-70 % HRmax) |
| 17 | Sphery Racer (20 min. competition mode) | Jogging (40 min. at 65-70 % HRmax) |
| 18 | Sphery Racer (20 min. competition mode) | Bicycle riding (45 min. at 65-70 % HRmax) |
| 19 | Sphery Racer (20 min. competition mode) | Jogging (35 min. at 65-70 % HRmax) |
| 20 | Sphery Racer (25 min. competition mode) | Jogging (45 min. at 65-70 % HRmax) |
| 21 | Sphery Racer (30 min. competition mode) | Bicycle riding (45 min. at 65-70 % HRmax) |
| 22 | Sphery Racer (30 min. competition mode) | Jogging (40 min. at 65-70 % HRmax) |
| 23 | Sphery Racer (30 min. competition mode) | Jogging (45 min. at 65-70 % HRmax) |
| 24 | Sphery Racer (30 min. competition mode) | Bicycle riding (45 min. at 65-70 % HRmax) |

*Note:* Each MICT session started with an addition 3-minute warm-up. Min = Minutes, HRmax = maximal heart rate. Competition mode consists of a continuous game session, without the usual 15-second breaks that follow the completion of each game level in non-competition mode.
