# Supplementary material for: Effects of exergaming versus endurance training on cardiorespiratory fitness and hemodynamic parameters: a randomized controlled trial
Source: Eur J Appl Physiol. 2025 Mar 11;125(7):1817–30. doi: 10.1007/s00421-025-05743-z (PMC12227349; doi:10.1007/s00421-025-05743-z)
Supplement: Supplementary file 2 — Supplementary file2 (DOCX 33 KB) [file 421_2025_5743_MOESM2_ESM.docx]

**Supplementary Material 3.** Fixed effects parameter estimates for the different outcome variables.

|  |  |  |  | **95% CI** | |  |  |  |
| --- | --- | --- | --- | --- | --- | --- | --- | --- |
|  | **Effect** | **Estimate** | **SE** | **Lower** | **Upper** | ***df*** | ***F*** | ***p*** |
| BMI (kg/m^2^) | Intercept | 23.99 | 0.34 | 23.33 | 24.65 | 39.21 | 71.6 | < .001 |
|  | Group | -0.91 | 0.68 | -2.24 | 0.42 | 41.12 | -1.34 | .188 |
|  | Time | -0.03 | 0.03 | -0.08 | 0.02 | 39.11 | -1.06 | .294 |
|  | Body Mass | 0.34 | 0.01 | 0.31 | 0.36 | 53.41 | 26.66 | < .001 |
|  | Group × Time | -0.02 | 0.05 | -0.13 | 0.08 | 38.53 | -0.45 | .652 |
| WHtR | Intercept | 0.45 | 0.01 | 0.44 | 0.46 | 40.04 | 79.27 | < .001 |
|  | Group | -0.01 | 0.01 | -0.03 | 0.02 | 40.34 | -0.42 | .678 |
|  | Time | 0 | 0.01 | -0.01 | 0.01 | 41.11 | 0.31 | .756 |
|  | Body Mass | 0 | 0 | 0 | 0 | 42.09 | 5.69 | < .001 |
|  | Group × Time | 0.01 | 0.01 | -0.02 | 0.03 | 41 | 0.77 | .445 |
| BF (%) | Intercept | 28.37 | 1.18 | 26.05 | 30.69 | 38.54 | 24.01 | < .001 |
|  | Group | -1.92 | 2.5 | -6.83 | 2.99 | 41.25 | -0.77 | .447 |
|  | Time | -1.22 | 0.33 | -1.86 | -0.59 | 39.9 | -3.76 | < .001 |
|  | Body Mass | 0.38 | 0.09 | 0.19 | 0.56 | 68.46 | 4.04 | < .001 |
|  | Group × Time | -0.81 | 0.65 | -2.08 | 0.45 | 38.95 | -1.26 | .215 |
| Mean HR (min^-1^) | Intercept | 66.33 | 1.11 | 64.15 | 68.5 | 40.02 | 59.73 | < .001 |
|  | Group | -0.03 | 2.4 | -4.74 | 4.67 | 40.5 | -0.01 | .989 |
|  | Time | -4.16 | 0.95 | -6.01 | -2.3 | 41.12 | -4.38 | < .001 |
|  | Body Mass | 0.05 | 0.1 | -0.15 | 0.25 | 43.5 | 0.46 | .647 |
|  | Group × Time | 4.74 | 1.89 | 1.03 | 8.45 | 40.94 | 2.5 | .016 |
| RMSSD (ms) | Intercept | 59.29 | 4.08 | 51.3 | 67.28 | 40.08 | 14.55 | < .001 |
|  | Group | 12.66 | 8.79 | -4.58 | 29.89 | 40.79 | 1.44 | .158 |
|  | Time | 11.4 | 2.83 | 5.86 | 16.95 | 41.23 | 4.03 | < .001 |
|  | Body Mass | -0.82 | 0.37 | -1.55 | -0.09 | 45.35 | -2.21 | .032 |
|  | Group × Time | -11.07 | 5.64 | -22.14 | -0.01 | 40.96 | -1.96 | .057 |
| SDNN (ms) | Intercept | 69.79 | 3.8 | 62.34 | 77.25 | 40.1 | 18.34 | < .001 |
|  | Group | 11.96 | 8.22 | -4.14 | 28.06 | 40.73 | 1.46 | .153 |
|  | Time | 12.96 | 2.81 | 7.45 | 18.48 | 41.23 | 4.61 | < .001 |
|  | Body Mass | -0.74 | 0.35 | -1.43 | -0.06 | 44.74 | -2.14 | .038 |
|  | Group × Time | 3.03 | 5.62 | -7.97 | 14.04 | 40.99 | 0.54 | .592 |
| MeanRR (ms) | Intercept | 925.13 | 17.07 | 891.67 | 958.59 | 40.07 | 54.19 | < .001 |
|  | Group | 13.77 | 36.86 | -58.47 | 86.02 | 40.7 | 0.37 | .711 |
|  | Time | 53.09 | 12.62 | 28.35 | 77.83 | 41.2 | 4.21 | < .001 |
|  | Body Mass | -0.24 | 1.56 | -3.3 | 2.82 | 44.71 | -0.15 | .881 |
|  | Group × Time | -44.93 | 25.21 | -94.33 | 4.48 | 40.96 | -1.78 | .082 |
| LF/HF | Intercept | 1.53 | 0.21 | 1.11 | 1.94 | 40.05 | 7.23 | < .001 |
|  | Group | -0.42 | 0.46 | -1.31 | 0.48 | 40.41 | -0.92 | .365 |
|  | Time | -0.32 | 0.21 | -0.73 | 0.09 | 41.13 | -1.54 | .131 |
|  | Body Mass | 0 | 0.02 | -0.04 | 0.03 | 42.63 | -0.23 | .821 |
|  | Group × Time | 0.72 | 0.42 | -0.1 | 1.54 | 40.99 | 1.73 | .092 |
| LF-Power (ms^2^) | Intercept | 1907.79 | 289.7 | 1339.99 | 2475.59 | 40 | 6.59 | < .001 |
|  | Group | 737.87 | 626.36 | -489.78 | 1965.52 | 40.43 | 1.18 | .246 |
|  | Time | 491.5 | 261.66 | -21.35 | 1004.36 | 41.09 | 1.88 | .067 |
|  | Body Mass | -60.89 | 26.74 | -113.29 | -8.49 | 43.11 | -2.28 | .028 |
|  | Group × Time | 692.5 | 522.78 | -332.13 | 1717.12 | 40.92 | 1.32 | .193 |
| HF-Power (ms^2^) | Intercept | 1920.96 | 370.58 | 1194.65 | 2647.28 | 40.17 | 5.18 | < .001 |
|  | Group | 1617.48 | 796.31 | 56.74 | 3178.21 | 41.47 | 2.03 | .049 |
|  | Time | 591.27 | 179.94 | 238.59 | 943.94 | 41.42 | 3.29 | .002 |
|  | Body Mass | -65.67 | 32.72 | -129.79 | -1.55 | 50.84 | -2.01 | .05 |
|  | Group × Time | -359.59 | 358.68 | -1062.59 | 343.41 | 40.94 | -1 | .322 |
| SBP (mmHg) | Intercept | 117.07 | 1.35 | 114.43 | 119.71 | 40.13 | 86.78 | < .001 |
|  | Group | -1.5 | 2.91 | -7.2 | 4.2 | 40.98 | -0.51 | .61 |
|  | Time | -4.65 | 0.84 | -6.3 | -3.01 | 41.3 | -5.55 | < .001 |
|  | Body Mass | 0.32 | 0.12 | 0.08 | 0.56 | 46.68 | 2.61 | .012 |
|  | Group × Time | 4.43 | 1.67 | 1.15 | 7.71 | 40.97 | 2.65 | .012 |
| DBP (mmHg) | Intercept | 73.06 | 1.22 | 70.67 | 75.44 | 40.12 | 60.03 | < .001 |
|  | Group | -1.41 | 2.63 | -6.56 | 3.74 | 40.84 | -0.54 | .595 |
|  | Time | -1.78 | 0.84 | -3.42 | -0.14 | 41.27 | -2.13 | .039 |
|  | Body Mass | 0.24 | 0.11 | 0.02 | 0.45 | 45.49 | 2.13 | .039 |
|  | Group × Time | 2.74 | 1.67 | -0.53 | 6.02 | 41 | 1.64 | .108 |
| HRmax (min^-1^) | Intercept | 185.42 | 1.66 | 182.17 | 188.67 | 40.15 | 111.78 | < .001 |
|  | Group | -0.46 | 3.57 | -7.47 | 6.54 | 41.1 | -0.13 | .898 |
|  | Time | -0.94 | 0.97 | -2.84 | 0.96 | 41.34 | -0.97 | .336 |
|  | Body Mass | 0.01 | 0.15 | -0.28 | 0.3 | 47.56 | 0.06 | .951 |
|  | Group × Time | 1.11 | 1.93 | -2.68 | 4.9 | 40.98 | 0.57 | .57 |
| VO_2_peak (mL/kg/min) | Intercept | 36.66 | 1.36 | 33.99 | 39.33 | 40 | 26.92 | < .001 |
|  | Group | -0.67 | 2.88 | -6.32 | 4.97 | 42.86 | -0.23 | .816 |
|  | Time | 2.25 | 0.35 | 1.55 | 2.94 | 41.35 | 6.34 | < .001 |
|  | Body Mass | -0.02 | 0.11 | -0.23 | 0.19 | 71.62 | -0.19 | .848 |
|  | Group × Time | -2.99 | 0.7 | -4.36 | -1.61 | 40.36 | -4.26 | < .001 |
| MVPA (min.) | Intercept | 145.99 | 18.23 | 110.26 | 181.71 | 40.05 | 8.01 | < .001 |
|  | Group | 17.34 | 39.43 | -59.94 | 94.61 | 40.41 | 0.44 | .662 |
|  | Time | 8.67 | 18.12 | -26.84 | 44.18 | 41.13 | 0.48 | .635 |
|  | Body Mass | 0.1 | 1.69 | -3.21 | 3.41 | 42.62 | 0.06 | .952 |
|  | Group × Time | -23.43 | 36.2 | -94.38 | 47.52 | 40.99 | -0.65 | .521 |
| PACES | Intercept | 65.86 | 1.21 | 63.5 | 68.22 | 40.07 | 54.62 | < .001 |
|  | Group | -6.06 | 2.61 | -11.17 | -0.95 | 40.48 | -2.32 | .025 |
|  | Time | -0.04 | 1.13 | -2.25 | 2.17 | 41.15 | -0.04 | .972 |
|  | Body Mass | 0.01 | 0.11 | -0.21 | 0.22 | 42.97 | 0.05 | .957 |
|  | Group × Time | -0.26 | 2.25 | -4.67 | 4.15 | 41 | -0.11 | .909 |

*Note.* SE = Standard error, CI = Confidence interval, BMI = Body Mass Index, WHtR = Waist-to-height-ratio, BF = Body fat, resting HR = Resting heart rate, RMSSD = Root mean square of successive differences between normal heartbeats, SDNN = Standard deviation of all normal-to-normal intervals, MeanRR = Average time interval between consecutive R-waves, HF-Power = High-frequency power, LF-Power = Low-frequency power, LF/HF = Ratio of LF- to HF-power, SBP = Systolic blood pressure, DBP = Diastolic blood pressure, HRmax = Heart rate max, VO_2_peak = Peak oxygen consumption, MVPA = Minutes of moderate to vigorous physical activity, PACES = Physical activity enjoyment.
