# Supplementary material for: Effects of exergaming versus endurance training on cardiorespiratory fitness and hemodynamic parameters: a randomized controlled trial
Source: Eur J Appl Physiol. 2025 Mar 11;125(7):1817–30. doi: 10.1007/s00421-025-05743-z (PMC12227349; doi:10.1007/s00421-025-05743-z)

**Supplemental Digital Content 4:** Overview of the statistical models

**Statistical Analyses**

**Mixed Model BMI**

| Model Info | | | |
| --- | --- | --- | --- |
| **Info** | |  | |
| Estimate |  | Linear mixed model fit by REML |  |
| Call |  | BMI_t1 ~ 1 + Gruppe + Time + Gewicht_t1kg + Gruppe:Time+( 1 \| ID ) |  |
| AIC |  | 168.20 |  |
| BIC |  | 201.31 |  |
| LogLikel. |  | -85.06 |  |
| R-squared Marginal |  | 0.74 |  |
| R-squared Conditional |  | 1.00 |  |
| Converged |  | yes |  |
| Optimizer |  | bobyqa |  |
|  | | | |

**Model Results**

| Fixed Effect Omnibus tests | | | | | | | | | |
| --- | --- | --- | --- | --- | --- | --- | --- | --- | --- |
|  | | **F** | | **Num df** | | **Den df** | | **p** | |
| Gruppe |  | 1.80 |  | 1 |  | 41.12 |  | 0.188 |  |
| Time |  | 1.13 |  | 1 |  | 39.11 |  | 0.294 |  |
| Gewicht_t1kg |  | 710.75 |  | 1 |  | 53.41 |  | < .001 |  |
| Gruppe ✻ Time |  | 0.21 |  | 1 |  | 38.53 |  | 0.652 |  |
| Anmerkung. Satterthwaite method for degrees of freedom | | | | | | | | | |
|  | | | | | | | | | |

| Fixed Effects Parameter Estimates | | | | | | | | | | | | | | | | | |
| --- | --- | --- | --- | --- | --- | --- | --- | --- | --- | --- | --- | --- | --- | --- | --- | --- | --- |
|  | | | | | | | | **95% Confidence Interval** | | | |  | | | | | |
| **Names** | | **Effect** | | **Estimate** | | **SE** | | **Lower** | | **Upper** | | **df** | | **t** | | **p** | |
| (Intercept) |  | (Intercept) |  | 23.99 |  | 0.34 |  | 23.33 |  | 24.65 |  | 39.21 |  | 71.60 |  | < .001 |  |
| Gruppe1 |  | 2 - 1 |  | -0.91 |  | 0.68 |  | -2.24 |  | 0.42 |  | 41.12 |  | -1.34 |  | 0.188 |  |
| Time1 |  | 2 - 1 |  | -0.03 |  | 0.03 |  | -0.08 |  | 0.02 |  | 39.11 |  | -1.06 |  | 0.294 |  |
| Gewicht_t1kg |  | Gewicht_t1kg |  | 0.34 |  | 0.01 |  | 0.31 |  | 0.36 |  | 53.41 |  | 26.66 |  | < .001 |  |
| Gruppe1 ✻ Time1 |  | 2 - 1 ✻ 2 - 1 |  | -0.02 |  | 0.05 |  | -0.13 |  | 0.08 |  | 38.53 |  | -0.45 |  | 0.652 |  |
|  | | | | | | | | | | | | | | | | | |

| Random Components | | | | | | | | | |
| --- | --- | --- | --- | --- | --- | --- | --- | --- | --- |
| **Groups** | | **Name** | | **SD** | | **Variance** | | **ICC** | |
| ID |  | (Intercept) |  | 2.19 |  | 4.82 |  | 1.00 |  |
| Residual |  |  |  | 0.12 |  | 0.01 |  |  |  |
| Anmerkung. Number of Obs: 86 , groups: ID 43 | | | | | | | | | |
|  | | | | | | | | | |

**Post Hoc Tests**

| Post Hoc Comparisons - Gruppe ✻ Time | | | | | | | | | | | | | | | | | | | |
| --- | --- | --- | --- | --- | --- | --- | --- | --- | --- | --- | --- | --- | --- | --- | --- | --- | --- | --- | --- |
| **Comparison** | | | | | | | | | |  | | | | | | | | | |
| **Gruppe** | | **Time** | |  | | **Gruppe** | | **Time** | | **Difference** | | **SE** | | **t** | | **df** | | **p_bonferroni_** | |
| 1 |  | 1 |  | - |  | 1 |  | 2 |  | 0.02 |  | 0.04 |  | 0.45 |  | 40.28 |  | 1.000 |  |
| 1 |  | 1 |  | - |  | 2 |  | 1 |  | 0.90 |  | 0.68 |  | 1.32 |  | 42.75 |  | 1.000 |  |
| 1 |  | 1 |  | - |  | 2 |  | 2 |  | 0.94 |  | 0.68 |  | 1.38 |  | 42.55 |  | 1.000 |  |
| 1 |  | 2 |  | - |  | 2 |  | 2 |  | 0.92 |  | 0.68 |  | 1.36 |  | 42.72 |  | 1.000 |  |
| 2 |  | 1 |  | - |  | 1 |  | 2 |  | -0.88 |  | 0.68 |  | -1.30 |  | 42.93 |  | 1.000 |  |
| 2 |  | 1 |  | - |  | 2 |  | 2 |  | 0.04 |  | 0.04 |  | 1.06 |  | 40.36 |  | 1.000 |  |
|  | | | | | | | | | | | | | | | | | | | |

**Estimated Marginal Means**

| Gruppe | | | | | | | | | | | |
| --- | --- | --- | --- | --- | --- | --- | --- | --- | --- | --- | --- |
|  | | | | | | | | **95% Confidence Interval** | | | |
| **Gruppe** | | **Mean** | | **SE** | | **df** | | **Lower** | | **Upper** | |
| 1 |  | 24.45 |  | 0.47 |  | 40.14 |  | 23.49 |  | 25.40 |  |
| 2 |  | 23.54 |  | 0.48 |  | 40.18 |  | 22.56 |  | 24.51 |  |
| Anmerkung. Estimated means are estimated averaging across interacting variables | | | | | | | | | | | |
|  | | | | | | | | | | | |

| Time | | | | | | | | | | | |
| --- | --- | --- | --- | --- | --- | --- | --- | --- | --- | --- | --- |
|  | | | | | | | | **95% Confidence Interval** | | | |
| **Time** | | **Mean** | | **SE** | | **df** | | **Lower** | | **Upper** | |
| 1 |  | 24.00 |  | 0.34 |  | 39.34 |  | 23.33 |  | 24.68 |  |
| 2 |  | 23.98 |  | 0.34 |  | 39.33 |  | 23.30 |  | 24.65 |  |
| Anmerkung. Estimated means are estimated averaging across interacting variables | | | | | | | | | | | |
|  | | | | | | | | | | | |

| Gruppe:Time | | | | | | | | | | | | | |
| --- | --- | --- | --- | --- | --- | --- | --- | --- | --- | --- | --- | --- | --- |
|  | | | | | | | | | | **95% Confidence Interval** | | | |
| **Gruppe** | | **Time** | | **Mean** | | **SE** | | **df** | | **Lower** | | **Upper** | |
| 1 |  | 1 |  | 24.45 |  | 0.47 |  | 40.18 |  | 23.50 |  | 25.41 |  |
| 2 |  | 1 |  | 23.56 |  | 0.48 |  | 40.41 |  | 22.58 |  | 24.53 |  |
| 1 |  | 2 |  | 24.44 |  | 0.47 |  | 40.35 |  | 23.48 |  | 25.39 |  |
| 2 |  | 2 |  | 23.52 |  | 0.48 |  | 40.21 |  | 22.54 |  | 24.49 |  |
| Anmerkung. Estimated means are estimated keeping constant other effects in the model to the mean | | | | | | | | | | | | | |
|  | | | | | | | | | | | | | |

**Effects Plots**


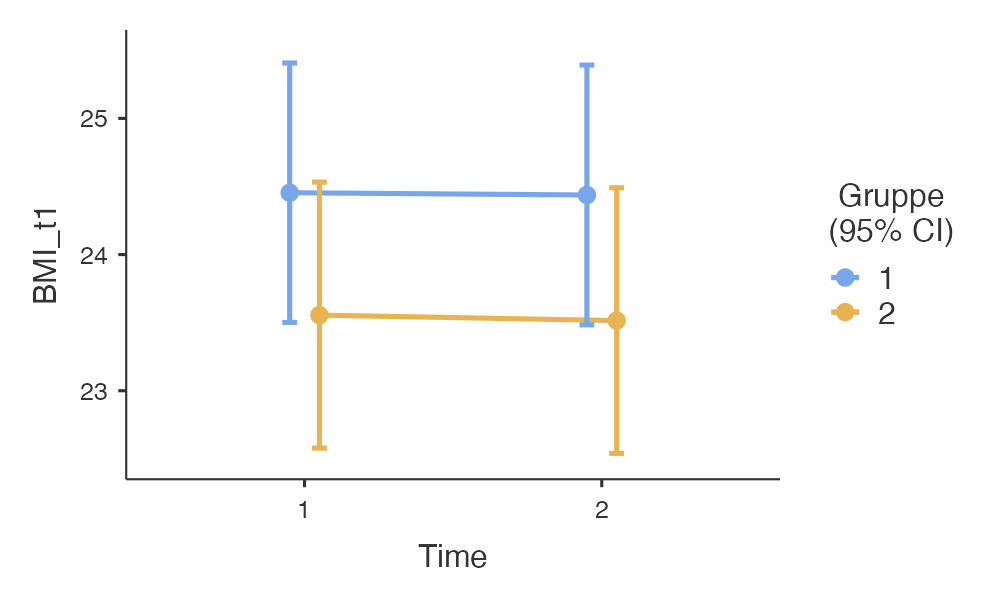


**Mixed Model WHTR**

| Model Info | | | |
| --- | --- | --- | --- |
| **Info** | |  | |
| Estimate |  | Linear mixed model fit by REML |  |
| Call |  | WHTR_t1 ~ 1 + Gruppe + Time + Gewicht_t1kg + Gruppe:Time+( 1 \| ID ) |  |
| AIC |  | -303.16 |  |
| BIC |  | -241.79 |  |
| LogLikel. |  | 136.48 |  |
| R-squared Marginal |  | 0.39 |  |
| R-squared Conditional |  | 0.71 |  |
| Converged |  | yes |  |
| Optimizer |  | bobyqa |  |
|  | | | |

**Model Results**

| Fixed Effect Omnibus tests | | | | | | | | | |
| --- | --- | --- | --- | --- | --- | --- | --- | --- | --- |
|  | | **F** | | **Num df** | | **Den df** | | **p** | |
| Gruppe |  | 0.17 |  | 1 |  | 40.34 |  | 0.678 |  |
| Time |  | 0.10 |  | 1 |  | 41.11 |  | 0.756 |  |
| Gewicht_t1kg |  | 32.37 |  | 1 |  | 42.09 |  | < .001 |  |
| Gruppe ✻ Time |  | 0.59 |  | 1 |  | 41.00 |  | 0.445 |  |
| Anmerkung. Satterthwaite method for degrees of freedom | | | | | | | | | |
|  | | | | | | | | | |

| Fixed Effects Parameter Estimates | | | | | | | | | | | | | | | | | |
| --- | --- | --- | --- | --- | --- | --- | --- | --- | --- | --- | --- | --- | --- | --- | --- | --- | --- |
|  | | | | | | | | **95% Confidence Interval** | | | |  | | | | | |
| **Names** | | **Effect** | | **Estimate** | | **SE** | | **Lower** | | **Upper** | | **df** | | **t** | | **p** | |
| (Intercept) |  | (Intercept) |  | 0.45 |  | 0.01 |  | 0.44 |  | 0.46 |  | 40.04 |  | 79.27 |  | < .001 |  |
| Gruppe1 |  | 2 - 1 |  | -0.01 |  | 0.01 |  | -0.03 |  | 0.02 |  | 40.34 |  | -0.42 |  | 0.678 |  |
| Time1 |  | 2 - 1 |  | 0.00 |  | 0.01 |  | -0.01 |  | 0.01 |  | 41.11 |  | 0.31 |  | 0.756 |  |
| Gewicht_t1kg |  | Gewicht_t1kg |  | 0.00 |  | 0.00 |  | 0.00 |  | 0.00 |  | 42.09 |  | 5.69 |  | < .001 |  |
| Gruppe1 ✻ Time1 |  | 2 - 1 ✻ 2 - 1 |  | 0.01 |  | 0.01 |  | -0.02 |  | 0.03 |  | 41.00 |  | 0.77 |  | 0.445 |  |
|  | | | | | | | | | | | | | | | | | |

| Random Components | | | | | | | | | |
| --- | --- | --- | --- | --- | --- | --- | --- | --- | --- |
| **Groups** | | **Name** | | **SD** | | **Variance** | | **ICC** | |
| ID |  | (Intercept) |  | 0.03 |  | 0.00 |  | 0.53 |  |
| Residual |  |  |  | 0.03 |  | 0.00 |  |  |  |
| Anmerkung. Number of Obs: 86 , groups: ID 43 | | | | | | | | | |
|  | | | | | | | | | |

**Post Hoc Tests**

| Post Hoc Comparisons - Gruppe ✻ Time | | | | | | | | | | | | | | | | | | | |
| --- | --- | --- | --- | --- | --- | --- | --- | --- | --- | --- | --- | --- | --- | --- | --- | --- | --- | --- | --- |
| **Comparison** | | | | | | | | | |  | | | | | | | | | |
| **Gruppe** | | **Time** | |  | | **Gruppe** | | **Time** | | **Difference** | | **SE** | | **t** | | **df** | | **p_bonferroni_** | |
| 1 |  | 1 |  | - |  | 1 |  | 2 |  | 0.00 |  | 0.01 |  | 0.33 |  | 41.00 |  | 1.000 |  |
| 1 |  | 1 |  | - |  | 2 |  | 1 |  | 0.01 |  | 0.01 |  | 0.72 |  | 60.14 |  | 1.000 |  |
| 1 |  | 1 |  | - |  | 2 |  | 2 |  | 0.00 |  | 0.01 |  | 0.23 |  | 60.39 |  | 1.000 |  |
| 1 |  | 2 |  | - |  | 2 |  | 2 |  | 0.00 |  | 0.01 |  | 0.02 |  | 60.18 |  | 1.000 |  |
| 2 |  | 1 |  | - |  | 1 |  | 2 |  | -0.01 |  | 0.01 |  | -0.51 |  | 59.93 |  | 1.000 |  |
| 2 |  | 1 |  | - |  | 2 |  | 2 |  | -0.01 |  | 0.01 |  | -0.76 |  | 41.01 |  | 1.000 |  |
|  | | | | | | | | | | | | | | | | | | | |

**Estimated Marginal Means**

| Gruppe | | | | | | | | | | | |
| --- | --- | --- | --- | --- | --- | --- | --- | --- | --- | --- | --- |
|  | | | | | | | | **95% Confidence Interval** | | | |
| **Gruppe** | | **Mean** | | **SE** | | **df** | | **Lower** | | **Upper** | |
| 1 |  | 0.45 |  | 0.01 |  | 40.20 |  | 0.44 |  | 0.47 |  |
| 2 |  | 0.45 |  | 0.01 |  | 40.21 |  | 0.43 |  | 0.47 |  |
| Anmerkung. Estimated means are estimated averaging across interacting variables | | | | | | | | | | | |
|  | | | | | | | | | | | |

| Time | | | | | | | | | | | |
| --- | --- | --- | --- | --- | --- | --- | --- | --- | --- | --- | --- |
|  | | | | | | | | **95% Confidence Interval** | | | |
| **Time** | | **Mean** | | **SE** | | **df** | | **Lower** | | **Upper** | |
| 1 |  | 0.45 |  | 0.01 |  | 62.74 |  | 0.44 |  | 0.46 |  |
| 2 |  | 0.45 |  | 0.01 |  | 62.76 |  | 0.44 |  | 0.47 |  |
| Anmerkung. Estimated means are estimated averaging across interacting variables | | | | | | | | | | | |
|  | | | | | | | | | | | |

| Gruppe:Time | | | | | | | | | | | | | |
| --- | --- | --- | --- | --- | --- | --- | --- | --- | --- | --- | --- | --- | --- |
|  | | | | | | | | | | **95% Confidence Interval** | | | |
| **Gruppe** | | **Time** | | **Mean** | | **SE** | | **df** | | **Lower** | | **Upper** | |
| 1 |  | 1 |  | 0.46 |  | 0.01 |  | 61.55 |  | 0.44 |  | 0.48 |  |
| 2 |  | 1 |  | 0.45 |  | 0.01 |  | 61.23 |  | 0.43 |  | 0.47 |  |
| 1 |  | 2 |  | 0.45 |  | 0.01 |  | 61.31 |  | 0.43 |  | 0.47 |  |
| 2 |  | 2 |  | 0.45 |  | 0.01 |  | 61.51 |  | 0.43 |  | 0.47 |  |
| Anmerkung. Estimated means are estimated keeping constant other effects in the model to the mean | | | | | | | | | | | | | |
|  | | | | | | | | | | | | | |

**Effects Plots**


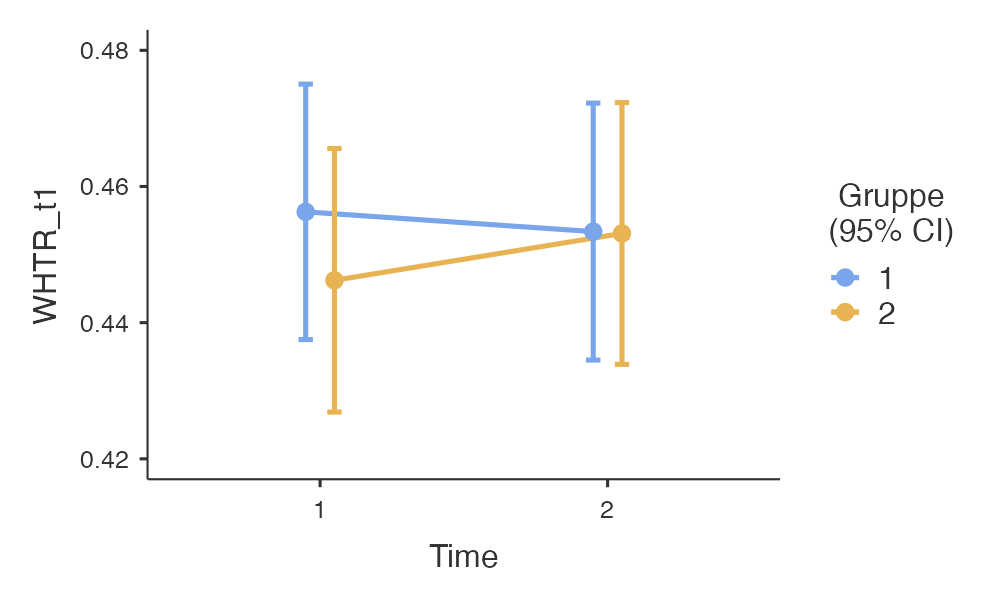


**Mixed Model Body Fat**

| Model Info | | | |
| --- | --- | --- | --- |
| **Info** | |  | |
| Estimate |  | Linear mixed model fit by REML |  |
| Call |  | KF_t1 ~ 1 + Gruppe + Time + Gewicht_t1kg + Gruppe:Time+( 1 \| ID ) |  |
| AIC |  | 493.50 |  |
| BIC |  | 507.47 |  |
| LogLikel. |  | -238.14 |  |
| R-squared Marginal |  | 0.22 |  |
| R-squared Conditional |  | 0.97 |  |
| Converged |  | yes |  |
| Optimizer |  | bobyqa |  |
|  | | | |

**Model Results**

| Fixed Effect Omnibus tests | | | | | | | | | |
| --- | --- | --- | --- | --- | --- | --- | --- | --- | --- |
|  | | **F** | | **Num df** | | **Den df** | | **p** | |
| Gruppe |  | 0.59 |  | 1 |  | 41.25 |  | 0.447 |  |
| Time |  | 14.12 |  | 1 |  | 39.90 |  | < .001 |  |
| Gewicht_t1kg |  | 16.31 |  | 1 |  | 68.46 |  | < .001 |  |
| Gruppe ✻ Time |  | 1.59 |  | 1 |  | 38.95 |  | 0.215 |  |
| Anmerkung. Satterthwaite method for degrees of freedom | | | | | | | | | |
|  | | | | | | | | | |

| Fixed Effects Parameter Estimates | | | | | | | | | | | | | | | | | |
| --- | --- | --- | --- | --- | --- | --- | --- | --- | --- | --- | --- | --- | --- | --- | --- | --- | --- |
|  | | | | | | | | **95% Confidence Interval** | | | |  | | | | | |
| **Names** | | **Effect** | | **Estimate** | | **SE** | | **Lower** | | **Upper** | | **df** | | **t** | | **p** | |
| (Intercept) |  | (Intercept) |  | 28.37 |  | 1.18 |  | 26.05 |  | 30.69 |  | 38.54 |  | 24.01 |  | < .001 |  |
| Gruppe1 |  | 2 - 1 |  | -1.92 |  | 2.50 |  | -6.83 |  | 2.99 |  | 41.25 |  | -0.77 |  | 0.447 |  |
| Time1 |  | 2 - 1 |  | -1.22 |  | 0.33 |  | -1.86 |  | -0.59 |  | 39.90 |  | -3.76 |  | < .001 |  |
| Gewicht_t1kg |  | Gewicht_t1kg |  | 0.38 |  | 0.09 |  | 0.19 |  | 0.56 |  | 68.46 |  | 4.04 |  | < .001 |  |
| Gruppe1 ✻ Time1 |  | 2 - 1 ✻ 2 - 1 |  | -0.81 |  | 0.65 |  | -2.08 |  | 0.45 |  | 38.95 |  | -1.26 |  | 0.215 |  |
|  | | | | | | | | | | | | | | | | | |

| Random Components | | | | | | | | | |
| --- | --- | --- | --- | --- | --- | --- | --- | --- | --- |
| **Groups** | | **Name** | | **SD** | | **Variance** | | **ICC** | |
| ID |  | (Intercept) |  | 7.67 |  | 58.89 |  | 0.96 |  |
| Residual |  |  |  | 1.50 |  | 2.24 |  |  |  |
| Anmerkung. Number of Obs: 86 , groups: ID 43 | | | | | | | | | |
|  | | | | | | | | | |

**Post Hoc Tests**

| Post Hoc Comparisons - Gruppe ✻ Time | | | | | | | | | | | | | | | | | | | |
| --- | --- | --- | --- | --- | --- | --- | --- | --- | --- | --- | --- | --- | --- | --- | --- | --- | --- | --- | --- |
| **Comparison** | | | | | | | | | |  | | | | | | | | | |
| **Gruppe** | | **Time** | |  | | **Gruppe** | | **Time** | | **Difference** | | **SE** | | **t** | | **df** | | **p_bonferroni_** | |
| 1 |  | 1 |  | - |  | 1 |  | 2 |  | 0.82 |  | 0.45 |  | 1.80 |  | 40.91 |  | 0.473 |  |
| 1 |  | 1 |  | - |  | 2 |  | 1 |  | 1.52 |  | 2.53 |  | 0.60 |  | 44.20 |  | 1.000 |  |
| 1 |  | 1 |  | - |  | 2 |  | 2 |  | 3.15 |  | 2.52 |  | 1.25 |  | 43.95 |  | 1.000 |  |
| 1 |  | 2 |  | - |  | 2 |  | 2 |  | 2.33 |  | 2.53 |  | 0.92 |  | 44.17 |  | 1.000 |  |
| 2 |  | 1 |  | - |  | 1 |  | 2 |  | -0.70 |  | 2.55 |  | -0.27 |  | 44.43 |  | 1.000 |  |
| 2 |  | 1 |  | - |  | 2 |  | 2 |  | 1.63 |  | 0.46 |  | 3.51 |  | 41.04 |  | 0.007 |  |
|  | | | | | | | | | | | | | | | | | | | |

**Estimated Marginal Means**

| Gruppe | | | | | | | | | | | |
| --- | --- | --- | --- | --- | --- | --- | --- | --- | --- | --- | --- |
|  | | | | | | | | **95% Confidence Interval** | | | |
| **Gruppe** | | **Mean** | | **SE** | | **df** | | **Lower** | | **Upper** | |
| 1 |  | 29.33 |  | 1.70 |  | 39.92 |  | 25.89 |  | 32.77 |  |
| 2 |  | 27.41 |  | 1.74 |  | 39.98 |  | 23.89 |  | 30.93 |  |
| Anmerkung. Estimated means are estimated averaging across interacting variables | | | | | | | | | | | |
|  | | | | | | | | | | | |

| Time | | | | | | | | | | | |
| --- | --- | --- | --- | --- | --- | --- | --- | --- | --- | --- | --- |
|  | | | | | | | | **95% Confidence Interval** | | | |
| **Time** | | **Mean** | | **SE** | | **df** | | **Lower** | | **Upper** | |
| 1 |  | 28.98 |  | 1.19 |  | 40.05 |  | 26.57 |  | 31.39 |  |
| 2 |  | 27.76 |  | 1.19 |  | 40.04 |  | 25.35 |  | 30.17 |  |
| Anmerkung. Estimated means are estimated averaging across interacting variables | | | | | | | | | | | |
|  | | | | | | | | | | | |

| Gruppe:Time | | | | | | | | | | | | | |
| --- | --- | --- | --- | --- | --- | --- | --- | --- | --- | --- | --- | --- | --- |
|  | | | | | | | | | | **95% Confidence Interval** | | | |
| **Gruppe** | | **Time** | | **Mean** | | **SE** | | **df** | | **Lower** | | **Upper** | |
| 1 |  | 1 |  | 29.74 |  | 1.71 |  | 41.24 |  | 26.28 |  | 33.19 |  |
| 2 |  | 1 |  | 28.22 |  | 1.76 |  | 41.57 |  | 24.66 |  | 31.78 |  |
| 1 |  | 2 |  | 28.92 |  | 1.72 |  | 41.49 |  | 25.45 |  | 32.40 |  |
| 2 |  | 2 |  | 26.59 |  | 1.75 |  | 41.29 |  | 23.05 |  | 30.13 |  |
| Anmerkung. Estimated means are estimated keeping constant other effects in the model to the mean | | | | | | | | | | | | | |
|  | | | | | | | | | | | | | |

**Effects Plots**


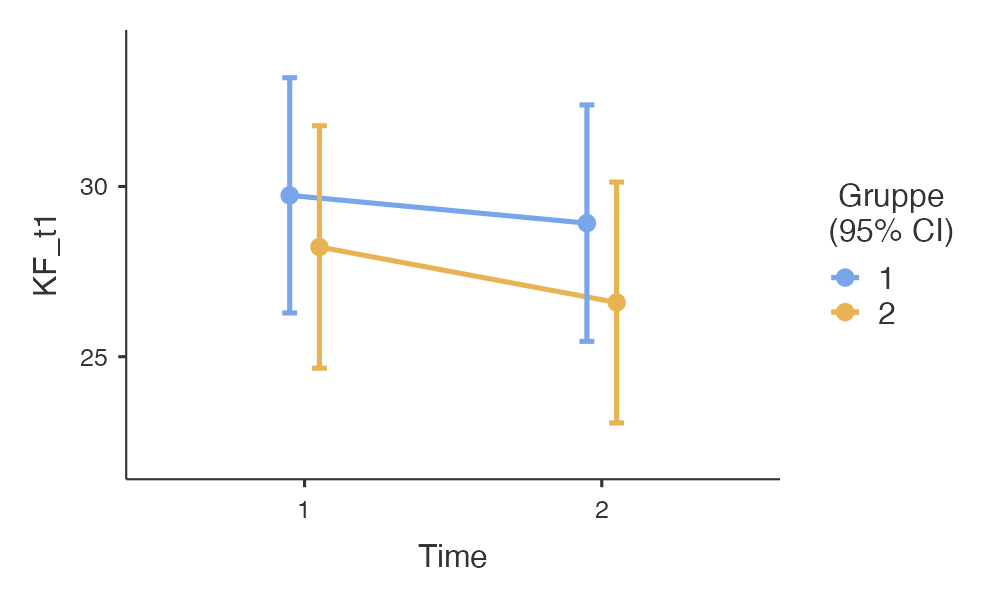


**Mixed Model Resting Heart Rate**

| Model Info | | | |
| --- | --- | --- | --- |
| **Info** | |  | |
| Estimate |  | Linear mixed model fit by REML |  |
| Call |  | HFMean_t1 ~ 1 + Gruppe + Time + Gewicht_t1kg + Gruppe:Time+( 1 \| ID ) |  |
| AIC |  | 580.62 |  |
| BIC |  | 590.36 |  |
| LogLikel. |  | -279.59 |  |
| R-squared Marginal |  | 0.09 |  |
| R-squared Conditional |  | 0.72 |  |
| Converged |  | yes |  |
| Optimizer |  | bobyqa |  |
|  | | | |

**Model Results**

| Fixed Effect Omnibus tests | | | | | | | | | |
| --- | --- | --- | --- | --- | --- | --- | --- | --- | --- |
|  | | **F** | | **Num df** | | **Den df** | | **p** | |
| Gruppe |  | 0.00 |  | 1 |  | 40.50 |  | 0.989 |  |
| Time |  | 19.22 |  | 1 |  | 41.12 |  | < .001 |  |
| Gewicht_t1kg |  | 0.21 |  | 1 |  | 43.50 |  | 0.647 |  |
| Gruppe ✻ Time |  | 6.26 |  | 1 |  | 40.94 |  | 0.016 |  |
| Anmerkung. Satterthwaite method for degrees of freedom | | | | | | | | | |
|  | | | | | | | | | |

| Fixed Effects Parameter Estimates | | | | | | | | | | | | | | | | | |
| --- | --- | --- | --- | --- | --- | --- | --- | --- | --- | --- | --- | --- | --- | --- | --- | --- | --- |
|  | | | | | | | | **95% Confidence Interval** | | | |  | | | | | |
| **Names** | | **Effect** | | **Estimate** | | **SE** | | **Lower** | | **Upper** | | **df** | | **t** | | **p** | |
| (Intercept) |  | (Intercept) |  | 66.33 |  | 1.11 |  | 64.15 |  | 68.50 |  | 40.02 |  | 59.73 |  | < .001 |  |
| Gruppe1 |  | 2 - 1 |  | -0.03 |  | 2.40 |  | -4.74 |  | 4.67 |  | 40.50 |  | -0.01 |  | 0.989 |  |
| Time1 |  | 2 - 1 |  | -4.16 |  | 0.95 |  | -6.01 |  | -2.30 |  | 41.12 |  | -4.38 |  | < .001 |  |
| Gewicht_t1kg |  | Gewicht_t1kg |  | 0.05 |  | 0.10 |  | -0.15 |  | 0.25 |  | 43.50 |  | 0.46 |  | 0.647 |  |
| Gruppe1 ✻ Time1 |  | 2 - 1 ✻ 2 - 1 |  | 4.74 |  | 1.89 |  | 1.03 |  | 8.45 |  | 40.94 |  | 2.50 |  | 0.016 |  |
|  | | | | | | | | | | | | | | | | | |

| Random Components | | | | | | | | | |
| --- | --- | --- | --- | --- | --- | --- | --- | --- | --- |
| **Groups** | | **Name** | | **SD** | | **Variance** | | **ICC** | |
| ID |  | (Intercept) |  | 6.58 |  | 43.36 |  | 0.69 |  |
| Residual |  |  |  | 4.39 |  | 19.26 |  |  |  |
| Anmerkung. Number of Obs: 86 , groups: ID 43 | | | | | | | | | |
|  | | | | | | | | | |

**Post Hoc Tests**

| Post Hoc Comparisons - Gruppe ✻ Time | | | | | | | | | | | | | | | | | | | |
| --- | --- | --- | --- | --- | --- | --- | --- | --- | --- | --- | --- | --- | --- | --- | --- | --- | --- | --- | --- |
| **Comparison** | | | | | | | | | |  | | | | | | | | | |
| **Gruppe** | | **Time** | |  | | **Gruppe** | | **Time** | | **Difference** | | **SE** | | **t** | | **df** | | **p_bonferroni_** | |
| 1 |  | 1 |  | - |  | 1 |  | 2 |  | 6.53 |  | 1.32 |  | 4.93 |  | 41.00 |  | < .001 |  |
| 1 |  | 1 |  | - |  | 2 |  | 1 |  | 2.40 |  | 2.58 |  | 0.93 |  | 52.71 |  | 1.000 |  |
| 1 |  | 1 |  | - |  | 2 |  | 2 |  | 4.19 |  | 2.57 |  | 1.63 |  | 52.85 |  | 0.651 |  |
| 1 |  | 2 |  | - |  | 2 |  | 2 |  | -2.34 |  | 2.58 |  | -0.91 |  | 52.73 |  | 1.000 |  |
| 2 |  | 1 |  | - |  | 1 |  | 2 |  | 4.12 |  | 2.60 |  | 1.59 |  | 52.60 |  | 0.710 |  |
| 2 |  | 1 |  | - |  | 2 |  | 2 |  | 1.79 |  | 1.36 |  | 1.32 |  | 41.02 |  | 1.000 |  |
|  | | | | | | | | | | | | | | | | | | | |

**Estimated Marginal Means**

| Gruppe | | | | | | | | | | | |
| --- | --- | --- | --- | --- | --- | --- | --- | --- | --- | --- | --- |
|  | | | | | | | | **95% Confidence Interval** | | | |
| **Gruppe** | | **Mean** | | **SE** | | **df** | | **Lower** | | **Upper** | |
| 1 |  | 66.34 |  | 1.61 |  | 40.27 |  | 63.08 |  | 69.60 |  |
| 2 |  | 66.31 |  | 1.66 |  | 40.28 |  | 62.96 |  | 69.65 |  |
| Anmerkung. Estimated means are estimated averaging across interacting variables | | | | | | | | | | | |
|  | | | | | | | | | | | |

| Time | | | | | | | | | | | |
| --- | --- | --- | --- | --- | --- | --- | --- | --- | --- | --- | --- |
|  | | | | | | | | **95% Confidence Interval** | | | |
| **Time** | | **Mean** | | **SE** | | **df** | | **Lower** | | **Upper** | |
| 1 |  | 68.40 |  | 1.21 |  | 54.14 |  | 65.98 |  | 70.82 |  |
| 2 |  | 64.25 |  | 1.21 |  | 54.15 |  | 61.83 |  | 66.67 |  |
| Anmerkung. Estimated means are estimated averaging across interacting variables | | | | | | | | | | | |
|  | | | | | | | | | | | |

| Gruppe:Time | | | | | | | | | | | | | |
| --- | --- | --- | --- | --- | --- | --- | --- | --- | --- | --- | --- | --- | --- |
|  | | | | | | | | | | **95% Confidence Interval** | | | |
| **Gruppe** | | **Time** | | **Mean** | | **SE** | | **df** | | **Lower** | | **Upper** | |
| 1 |  | 1 |  | 69.60 |  | 1.74 |  | 53.48 |  | 66.12 |  | 73.09 |  |
| 2 |  | 1 |  | 67.20 |  | 1.80 |  | 53.30 |  | 63.60 |  | 70.80 |  |
| 1 |  | 2 |  | 63.08 |  | 1.75 |  | 53.34 |  | 59.57 |  | 66.59 |  |
| 2 |  | 2 |  | 65.42 |  | 1.78 |  | 53.45 |  | 61.84 |  | 68.99 |  |
| Anmerkung. Estimated means are estimated keeping constant other effects in the model to the mean | | | | | | | | | | | | | |
|  | | | | | | | | | | | | | |

**Effects Plots**


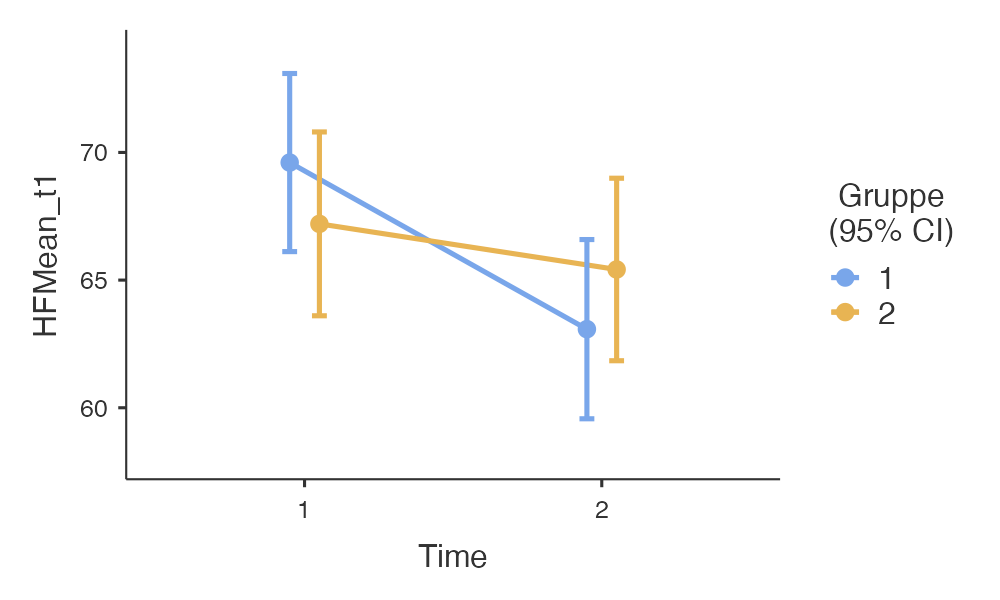


**Mixed Model RMSSD**

| Model Info | | | |
| --- | --- | --- | --- |
| **Info** | |  | |
| Estimate |  | Linear mixed model fit by REML |  |
| Call |  | RMSSD_t1 ~ 1 + Gruppe + Time + Gewicht_t1kg + Gruppe:Time+( 1 \| ID ) |  |
| AIC |  | 786.37 |  |
| BIC |  | 783.96 |  |
| LogLikel. |  | -376.39 |  |
| R-squared Marginal |  | 0.14 |  |
| R-squared Conditional |  | 0.82 |  |
| Converged |  | yes |  |
| Optimizer |  | bobyqa |  |
|  | | | |

**Model Results**

| Fixed Effect Omnibus tests | | | | | | | | | |
| --- | --- | --- | --- | --- | --- | --- | --- | --- | --- |
|  | | **F** | | **Num df** | | **Den df** | | **p** | |
| Gruppe |  | 2.07 |  | 1 |  | 40.79 |  | 0.158 |  |
| Time |  | 16.27 |  | 1 |  | 41.23 |  | < .001 |  |
| Gewicht_t1kg |  | 4.88 |  | 1 |  | 45.35 |  | 0.032 |  |
| Gruppe ✻ Time |  | 3.85 |  | 1 |  | 40.96 |  | 0.057 |  |
| Anmerkung. Satterthwaite method for degrees of freedom | | | | | | | | | |
|  | | | | | | | | | |

| Fixed Effects Parameter Estimates | | | | | | | | | | | | | | | | | |
| --- | --- | --- | --- | --- | --- | --- | --- | --- | --- | --- | --- | --- | --- | --- | --- | --- | --- |
|  | | | | | | | | **95% Confidence Interval** | | | |  | | | | | |
| **Names** | | **Effect** | | **Estimate** | | **SE** | | **Lower** | | **Upper** | | **df** | | **t** | | **p** | |
| (Intercept) |  | (Intercept) |  | 59.29 |  | 4.08 |  | 51.30 |  | 67.28 |  | 40.08 |  | 14.55 |  | < .001 |  |
| Gruppe1 |  | 2 - 1 |  | 12.66 |  | 8.79 |  | -4.58 |  | 29.89 |  | 40.79 |  | 1.44 |  | 0.158 |  |
| Time1 |  | 2 - 1 |  | 11.40 |  | 2.83 |  | 5.86 |  | 16.95 |  | 41.23 |  | 4.03 |  | < .001 |  |
| Gewicht_t1kg |  | Gewicht_t1kg |  | -0.82 |  | 0.37 |  | -1.55 |  | -0.09 |  | 45.35 |  | -2.21 |  | 0.032 |  |
| Gruppe1 ✻ Time1 |  | 2 - 1 ✻ 2 - 1 |  | -11.07 |  | 5.64 |  | -22.14 |  | -0.01 |  | 40.96 |  | -1.96 |  | 0.057 |  |
|  | | | | | | | | | | | | | | | | | |

| Random Components | | | | | | | | | |
| --- | --- | --- | --- | --- | --- | --- | --- | --- | --- |
| **Groups** | | **Name** | | **SD** | | **Variance** | | **ICC** | |
| ID |  | (Intercept) |  | 25.06 |  | 628.08 |  | 0.79 |  |
| Residual |  |  |  | 13.08 |  | 171.17 |  |  |  |
| Anmerkung. Number of Obs: 86 , groups: ID 43 | | | | | | | | | |
|  | | | | | | | | | |

**Post Hoc Tests**

| Post Hoc Comparisons - Gruppe ✻ Time | | | | | | | | | | | | | | | | | | | |
| --- | --- | --- | --- | --- | --- | --- | --- | --- | --- | --- | --- | --- | --- | --- | --- | --- | --- | --- | --- |
| **Comparison** | | | | | | | | | |  | | | | | | | | | |
| **Gruppe** | | **Time** | |  | | **Gruppe** | | **Time** | | **Difference** | | **SE** | | **t** | | **df** | | **p_bonferroni_** | |
| 1 |  | 1 |  | - |  | 1 |  | 2 |  | -16.94 |  | 3.95 |  | -4.29 |  | 41.00 |  | < .001 |  |
| 1 |  | 1 |  | - |  | 2 |  | 1 |  | -18.19 |  | 9.25 |  | -1.97 |  | 48.93 |  | 0.329 |  |
| 1 |  | 1 |  | - |  | 2 |  | 2 |  | -24.06 |  | 9.18 |  | -2.62 |  | 48.99 |  | 0.070 |  |
| 1 |  | 2 |  | - |  | 2 |  | 2 |  | -7.12 |  | 9.24 |  | -0.77 |  | 48.94 |  | 1.000 |  |
| 2 |  | 1 |  | - |  | 1 |  | 2 |  | 1.25 |  | 9.31 |  | 0.13 |  | 48.89 |  | 1.000 |  |
| 2 |  | 1 |  | - |  | 2 |  | 2 |  | -5.87 |  | 4.04 |  | -1.45 |  | 41.03 |  | 0.926 |  |
|  | | | | | | | | | | | | | | | | | | | |

**Estimated Marginal Means**

| Gruppe | | | | | | | | | | | |
| --- | --- | --- | --- | --- | --- | --- | --- | --- | --- | --- | --- |
|  | | | | | | | | **95% Confidence Interval** | | | |
| **Gruppe** | | **Mean** | | **SE** | | **df** | | **Lower** | | **Upper** | |
| 1 |  | 52.96 |  | 5.92 |  | 40.45 |  | 41.00 |  | 64.92 |  |
| 2 |  | 65.62 |  | 6.07 |  | 40.47 |  | 53.36 |  | 77.88 |  |
| Anmerkung. Estimated means are estimated averaging across interacting variables | | | | | | | | | | | |
|  | | | | | | | | | | | |

| Time | | | | | | | | | | | |
| --- | --- | --- | --- | --- | --- | --- | --- | --- | --- | --- | --- |
|  | | | | | | | | **95% Confidence Interval** | | | |
| **Time** | | **Mean** | | **SE** | | **df** | | **Lower** | | **Upper** | |
| 1 |  | 53.59 |  | 4.31 |  | 49.57 |  | 44.92 |  | 62.26 |  |
| 2 |  | 64.99 |  | 4.31 |  | 49.57 |  | 56.33 |  | 73.66 |  |
| Anmerkung. Estimated means are estimated averaging across interacting variables | | | | | | | | | | | |
|  | | | | | | | | | | | |

| Gruppe:Time | | | | | | | | | | | | | |
| --- | --- | --- | --- | --- | --- | --- | --- | --- | --- | --- | --- | --- | --- |
|  | | | | | | | | | | **95% Confidence Interval** | | | |
| **Gruppe** | | **Time** | | **Mean** | | **SE** | | **df** | | **Lower** | | **Upper** | |
| 1 |  | 1 |  | 44.49 |  | 6.22 |  | 49.30 |  | 31.99 |  | 56.99 |  |
| 2 |  | 1 |  | 62.69 |  | 6.42 |  | 49.23 |  | 49.78 |  | 75.59 |  |
| 1 |  | 2 |  | 61.43 |  | 6.26 |  | 49.25 |  | 48.86 |  | 74.01 |  |
| 2 |  | 2 |  | 68.55 |  | 6.37 |  | 49.29 |  | 55.75 |  | 81.36 |  |
| Anmerkung. Estimated means are estimated keeping constant other effects in the model to the mean | | | | | | | | | | | | | |
|  | | | | | | | | | | | | | |

**Effects Plots**


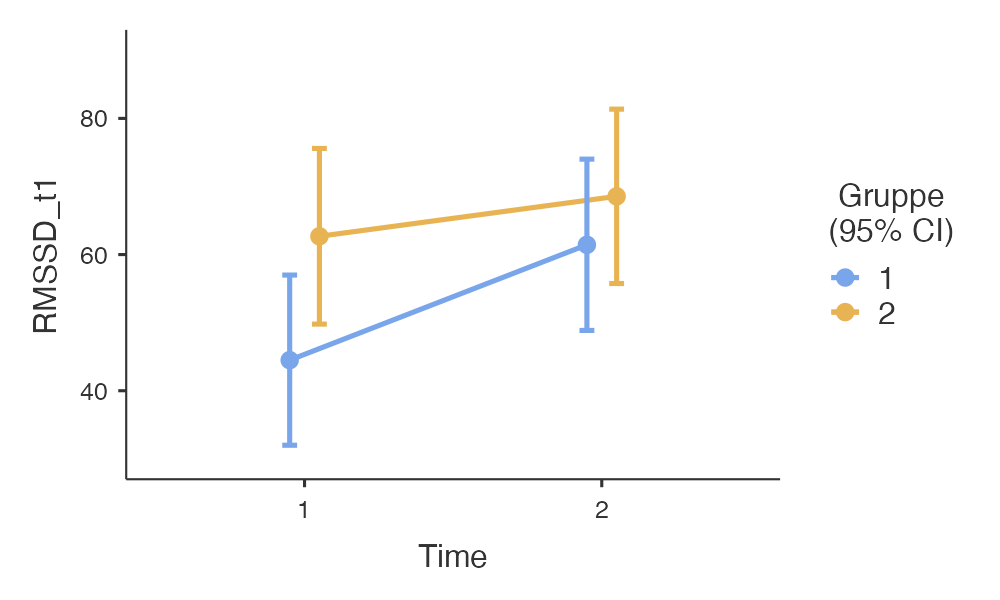


**Mixed Model SDNN**

| Model Info | | | |
| --- | --- | --- | --- |
| **Info** | |  | |
| Estimate |  | Linear mixed model fit by REML |  |
| Call |  | SDNN_t1 ~ 1 + Gruppe + Time + Gewicht_t1kg + Gruppe:Time+( 1 \| ID ) |  |
| AIC |  | 780.03 |  |
| BIC |  | 778.04 |  |
| LogLikel. |  | -373.43 |  |
| R-squared Marginal |  | 0.14 |  |
| R-squared Conditional |  | 0.79 |  |
| Converged |  | yes |  |
| Optimizer |  | bobyqa |  |
|  | | | |

**Model Results**

| Fixed Effect Omnibus tests | | | | | | | | | |
| --- | --- | --- | --- | --- | --- | --- | --- | --- | --- |
|  | | **F** | | **Num df** | | **Den df** | | **p** | |
| Gruppe |  | 2.12 |  | 1 |  | 40.73 |  | 0.153 |  |
| Time |  | 21.25 |  | 1 |  | 41.23 |  | < .001 |  |
| Gewicht_t1kg |  | 4.57 |  | 1 |  | 44.74 |  | 0.038 |  |
| Gruppe ✻ Time |  | 0.29 |  | 1 |  | 40.99 |  | 0.592 |  |
| Anmerkung. Satterthwaite method for degrees of freedom | | | | | | | | | |
|  | | | | | | | | | |

| Fixed Effects Parameter Estimates | | | | | | | | | | | | | | | | | |
| --- | --- | --- | --- | --- | --- | --- | --- | --- | --- | --- | --- | --- | --- | --- | --- | --- | --- |
|  | | | | | | | | **95% Confidence Interval** | | | |  | | | | | |
| **Names** | | **Effect** | | **Estimate** | | **SE** | | **Lower** | | **Upper** | | **df** | | **t** | | **p** | |
| (Intercept) |  | (Intercept) |  | 69.79 |  | 3.80 |  | 62.34 |  | 77.25 |  | 40.10 |  | 18.34 |  | < .001 |  |
| Gruppe1 |  | 2 - 1 |  | 11.96 |  | 8.22 |  | -4.14 |  | 28.06 |  | 40.73 |  | 1.46 |  | 0.153 |  |
| Time1 |  | 2 - 1 |  | 12.96 |  | 2.81 |  | 7.45 |  | 18.48 |  | 41.23 |  | 4.61 |  | < .001 |  |
| Gewicht_t1kg |  | Gewicht_t1kg |  | -0.74 |  | 0.35 |  | -1.43 |  | -0.06 |  | 44.74 |  | -2.14 |  | 0.038 |  |
| Gruppe1 ✻ Time1 |  | 2 - 1 ✻ 2 - 1 |  | 3.03 |  | 5.62 |  | -7.97 |  | 14.04 |  | 40.99 |  | 0.54 |  | 0.592 |  |
|  | | | | | | | | | | | | | | | | | |

| Random Components | | | | | | | | | |
| --- | --- | --- | --- | --- | --- | --- | --- | --- | --- |
| **Groups** | | **Name** | | **SD** | | **Variance** | | **ICC** | |
| ID |  | (Intercept) |  | 23.18 |  | 537.36 |  | 0.76 |  |
| Residual |  |  |  | 13.02 |  | 169.46 |  |  |  |
| Anmerkung. Number of Obs: 86 , groups: ID 43 | | | | | | | | | |
|  | | | | | | | | | |

**Post Hoc Tests**

| Post Hoc Comparisons - Gruppe ✻ Time | | | | | | | | | | | | | | | | | | | |
| --- | --- | --- | --- | --- | --- | --- | --- | --- | --- | --- | --- | --- | --- | --- | --- | --- | --- | --- | --- |
| **Comparison** | | | | | | | | | |  | | | | | | | | | |
| **Gruppe** | | **Time** | |  | | **Gruppe** | | **Time** | | **Difference** | | **SE** | | **t** | | **df** | | **p_bonferroni_** | |
| 1 |  | 1 |  | - |  | 1 |  | 2 |  | -11.45 |  | 3.93 |  | -2.91 |  | 41.00 |  | 0.034 |  |
| 1 |  | 1 |  | - |  | 2 |  | 1 |  | -10.44 |  | 8.69 |  | -1.20 |  | 49.92 |  | 1.000 |  |
| 1 |  | 1 |  | - |  | 2 |  | 2 |  | -24.92 |  | 8.63 |  | -2.89 |  | 50.00 |  | 0.034 |  |
| 1 |  | 2 |  | - |  | 2 |  | 2 |  | -13.48 |  | 8.68 |  | -1.55 |  | 49.94 |  | 0.762 |  |
| 2 |  | 1 |  | - |  | 1 |  | 2 |  | -1.00 |  | 8.75 |  | -0.11 |  | 49.86 |  | 1.000 |  |
| 2 |  | 1 |  | - |  | 2 |  | 2 |  | -14.48 |  | 4.02 |  | -3.60 |  | 41.03 |  | 0.005 |  |
|  | | | | | | | | | | | | | | | | | | | |

**Estimated Marginal Means**

| Gruppe | | | | | | | | | | | |
| --- | --- | --- | --- | --- | --- | --- | --- | --- | --- | --- | --- |
|  | | | | | | | | **95% Confidence Interval** | | | |
| **Gruppe** | | **Mean** | | **SE** | | **df** | | **Lower** | | **Upper** | |
| 1 |  | 63.81 |  | 5.53 |  | 40.43 |  | 52.64 |  | 74.98 |  |
| 2 |  | 75.77 |  | 5.67 |  | 40.44 |  | 64.32 |  | 87.23 |  |
| Anmerkung. Estimated means are estimated averaging across interacting variables | | | | | | | | | | | |
|  | | | | | | | | | | | |

| Time | | | | | | | | | | | |
| --- | --- | --- | --- | --- | --- | --- | --- | --- | --- | --- | --- |
|  | | | | | | | | **95% Confidence Interval** | | | |
| **Time** | | **Mean** | | **SE** | | **df** | | **Lower** | | **Upper** | |
| 1 |  | 63.31 |  | 4.06 |  | 50.82 |  | 55.17 |  | 71.46 |  |
| 2 |  | 76.28 |  | 4.06 |  | 50.82 |  | 68.13 |  | 84.42 |  |
| Anmerkung. Estimated means are estimated averaging across interacting variables | | | | | | | | | | | |
|  | | | | | | | | | | | |

| Gruppe:Time | | | | | | | | | | | | | |
| --- | --- | --- | --- | --- | --- | --- | --- | --- | --- | --- | --- | --- | --- |
|  | | | | | | | | | | **95% Confidence Interval** | | | |
| **Gruppe** | | **Time** | | **Mean** | | **SE** | | **df** | | **Lower** | | **Upper** | |
| 1 |  | 1 |  | 58.09 |  | 5.85 |  | 50.44 |  | 46.34 |  | 69.83 |  |
| 2 |  | 1 |  | 68.53 |  | 6.04 |  | 50.34 |  | 56.41 |  | 80.66 |  |
| 1 |  | 2 |  | 69.54 |  | 5.89 |  | 50.36 |  | 57.72 |  | 81.36 |  |
| 2 |  | 2 |  | 83.01 |  | 5.99 |  | 50.43 |  | 70.98 |  | 95.05 |  |
| Anmerkung. Estimated means are estimated keeping constant other effects in the model to the mean | | | | | | | | | | | | | |
|  | | | | | | | | | | | | | |

**Effects Plots**


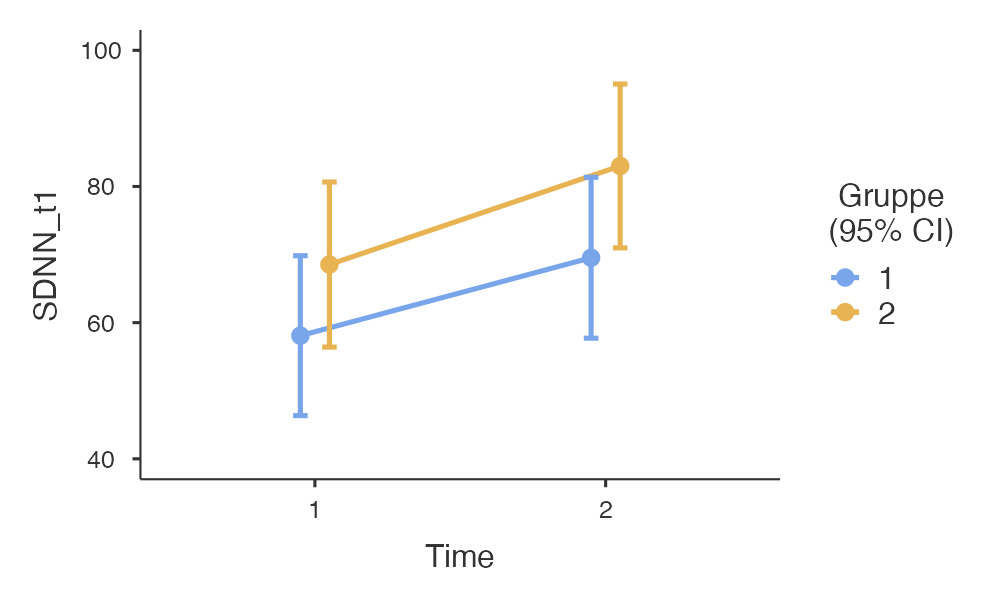


**Mixed Model MeanRR**

| Model Info | | | |
| --- | --- | --- | --- |
| **Info** | |  | |
| Estimate |  | Linear mixed model fit by REML |  |
| Call |  | MeanRR_t1 ~ 1 + Gruppe + Time + Gewicht_t1kg + Gruppe:Time+( 1 \| ID ) |  |
| AIC |  | 1038.25 |  |
| BIC |  | 1021.25 |  |
| LogLikel. |  | -495.04 |  |
| R-squared Marginal |  | 0.06 |  |
| R-squared Conditional |  | 0.77 |  |
| Converged |  | yes |  |
| Optimizer |  | bobyqa |  |
|  | | | |

**Model Results**

| Fixed Effect Omnibus tests | | | | | | | | | |
| --- | --- | --- | --- | --- | --- | --- | --- | --- | --- |
|  | | **F** | | **Num df** | | **Den df** | | **p** | |
| Gruppe |  | 0.14 |  | 1 |  | 40.70 |  | 0.711 |  |
| Time |  | 17.69 |  | 1 |  | 41.20 |  | < .001 |  |
| Gewicht_t1kg |  | 0.02 |  | 1 |  | 44.71 |  | 0.881 |  |
| Gruppe ✻ Time |  | 3.18 |  | 1 |  | 40.96 |  | 0.082 |  |
| Anmerkung. Satterthwaite method for degrees of freedom | | | | | | | | | |
|  | | | | | | | | | |

| Fixed Effects Parameter Estimates | | | | | | | | | | | | | | | | | |
| --- | --- | --- | --- | --- | --- | --- | --- | --- | --- | --- | --- | --- | --- | --- | --- | --- | --- |
|  | | | | | | | | **95% Confidence Interval** | | | |  | | | | | |
| **Names** | | **Effect** | | **Estimate** | | **SE** | | **Lower** | | **Upper** | | **df** | | **t** | | **p** | |
| (Intercept) |  | (Intercept) |  | 925.13 |  | 17.07 |  | 891.67 |  | 958.59 |  | 40.07 |  | 54.19 |  | < .001 |  |
| Gruppe1 |  | 2 - 1 |  | 13.77 |  | 36.86 |  | -58.47 |  | 86.02 |  | 40.70 |  | 0.37 |  | 0.711 |  |
| Time1 |  | 2 - 1 |  | 53.09 |  | 12.62 |  | 28.35 |  | 77.83 |  | 41.20 |  | 4.21 |  | < .001 |  |
| Gewicht_t1kg |  | Gewicht_t1kg |  | -0.24 |  | 1.56 |  | -3.30 |  | 2.82 |  | 44.71 |  | -0.15 |  | 0.881 |  |
| Gruppe1 ✻ Time1 |  | 2 - 1 ✻ 2 - 1 |  | -44.93 |  | 25.21 |  | -94.33 |  | 4.48 |  | 40.96 |  | -1.78 |  | 0.082 |  |
|  | | | | | | | | | | | | | | | | | |

| Random Components | | | | | | | | | |
| --- | --- | --- | --- | --- | --- | --- | --- | --- | --- |
| **Groups** | | **Name** | | **SD** | | **Variance** | | **ICC** | |
| ID |  | (Intercept) |  | 104.00 |  | 10816.01 |  | 0.76 |  |
| Residual |  |  |  | 58.43 |  | 3413.63 |  |  |  |
| Anmerkung. Number of Obs: 86 , groups: ID 43 | | | | | | | | | |
|  | | | | | | | | | |

**Post Hoc Tests**

| Post Hoc Comparisons - Gruppe ✻ Time | | | | | | | | | | | | | | | | | | | |
| --- | --- | --- | --- | --- | --- | --- | --- | --- | --- | --- | --- | --- | --- | --- | --- | --- | --- | --- | --- |
| **Comparison** | | | | | | | | | |  | | | | | | | | | |
| **Gruppe** | | **Time** | |  | | **Gruppe** | | **Time** | | **Difference** | | **SE** | | **t** | | **df** | | **p_bonferroni_** | |
| 1 |  | 1 |  | - |  | 1 |  | 2 |  | -75.56 |  | 17.63 |  | -4.29 |  | 41.00 |  | < .001 |  |
| 1 |  | 1 |  | - |  | 2 |  | 1 |  | -36.23 |  | 39.00 |  | -0.93 |  | 49.93 |  | 1.000 |  |
| 1 |  | 1 |  | - |  | 2 |  | 2 |  | -66.86 |  | 38.73 |  | -1.73 |  | 50.01 |  | 0.543 |  |
| 1 |  | 2 |  | - |  | 2 |  | 2 |  | 8.69 |  | 38.96 |  | 0.22 |  | 49.94 |  | 1.000 |  |
| 2 |  | 1 |  | - |  | 1 |  | 2 |  | -39.32 |  | 39.24 |  | -1.00 |  | 49.86 |  | 1.000 |  |
| 2 |  | 1 |  | - |  | 2 |  | 2 |  | -30.63 |  | 18.05 |  | -1.70 |  | 41.03 |  | 0.583 |  |
|  | | | | | | | | | | | | | | | | | | | |

**Estimated Marginal Means**

| Gruppe | | | | | | | | | | | |
| --- | --- | --- | --- | --- | --- | --- | --- | --- | --- | --- | --- |
|  | | | | | | | | **95% Confidence Interval** | | | |
| **Gruppe** | | **Mean** | | **SE** | | **df** | | **Lower** | | **Upper** | |
| 1 |  | 918.24 |  | 24.81 |  | 40.40 |  | 868.13 |  | 968.36 |  |
| 2 |  | 932.01 |  | 25.43 |  | 40.41 |  | 880.63 |  | 983.40 |  |
| Anmerkung. Estimated means are estimated averaging across interacting variables | | | | | | | | | | | |
|  | | | | | | | | | | | |

| Time | | | | | | | | | | | |
| --- | --- | --- | --- | --- | --- | --- | --- | --- | --- | --- | --- |
|  | | | | | | | | **95% Confidence Interval** | | | |
| **Time** | | **Mean** | | **SE** | | **df** | | **Lower** | | **Upper** | |
| 1 |  | 898.58 |  | 18.20 |  | 50.80 |  | 862.03 |  | 935.13 |  |
| 2 |  | 951.68 |  | 18.20 |  | 50.81 |  | 915.14 |  | 988.21 |  |
| Anmerkung. Estimated means are estimated averaging across interacting variables | | | | | | | | | | | |
|  | | | | | | | | | | | |

| Gruppe:Time | | | | | | | | | | | | | |
| --- | --- | --- | --- | --- | --- | --- | --- | --- | --- | --- | --- | --- | --- |
|  | | | | | | | | | | **95% Confidence Interval** | | | |
| **Gruppe** | | **Time** | | **Mean** | | **SE** | | **df** | | **Lower** | | **Upper** | |
| 1 |  | 1 |  | 880.46 |  | 26.24 |  | 50.42 |  | 827.77 |  | 933.16 |  |
| 2 |  | 1 |  | 916.70 |  | 27.09 |  | 50.32 |  | 862.30 |  | 971.10 |  |
| 1 |  | 2 |  | 956.02 |  | 26.41 |  | 50.35 |  | 902.99 |  | 1009.05 |  |
| 2 |  | 2 |  | 947.33 |  | 26.89 |  | 50.41 |  | 893.34 |  | 1001.32 |  |
| Anmerkung. Estimated means are estimated keeping constant other effects in the model to the mean | | | | | | | | | | | | | |
|  | | | | | | | | | | | | | |

**Effects Plots**


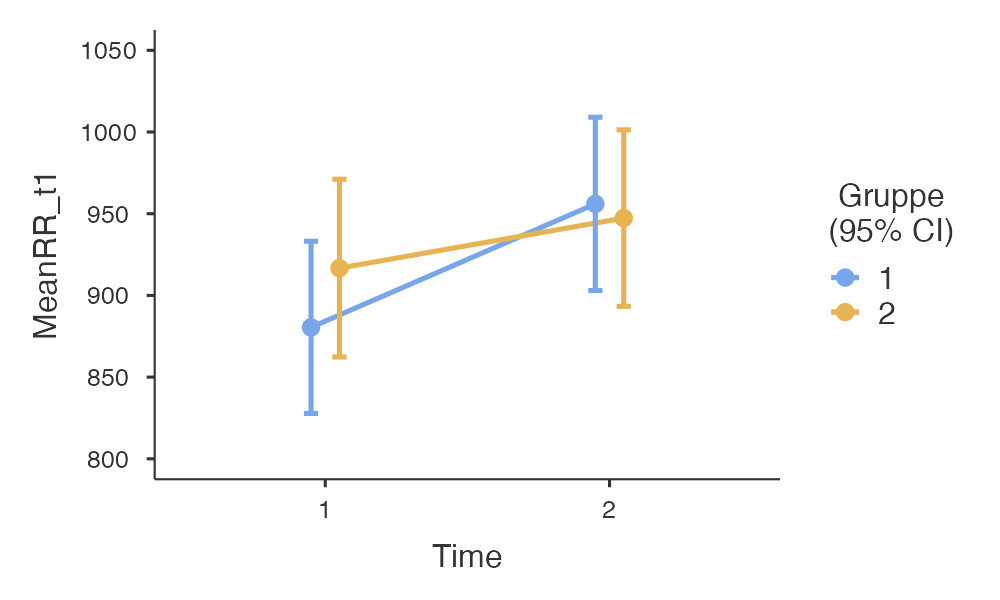


**Mixed Model LF/HF**

| Model Info | | | |
| --- | --- | --- | --- |
| **Info** | |  | |
| Estimate |  | Linear mixed model fit by REML |  |
| Call |  | LFHF_t1 ~ 1 + Gruppe + Time + Gewicht_t1kg + Gruppe:Time+( 1 \| ID ) |  |
| AIC |  | 307.90 |  |
| BIC |  | 333.63 |  |
| LogLikel. |  | -151.23 |  |
| R-squared Marginal |  | 0.05 |  |
| R-squared Conditional |  | 0.62 |  |
| Converged |  | yes |  |
| Optimizer |  | bobyqa |  |
|  | | | |

**Model Results**

| Fixed Effect Omnibus tests | | | | | | | | | |
| --- | --- | --- | --- | --- | --- | --- | --- | --- | --- |
|  | | **F** | | **Num df** | | **Den df** | | **p** | |
| Gruppe |  | 0.84 |  | 1 |  | 40.41 |  | 0.365 |  |
| Time |  | 2.38 |  | 1 |  | 41.13 |  | 0.131 |  |
| Gewicht_t1kg |  | 0.05 |  | 1 |  | 42.63 |  | 0.821 |  |
| Gruppe ✻ Time |  | 2.98 |  | 1 |  | 40.99 |  | 0.092 |  |
| Anmerkung. Satterthwaite method for degrees of freedom | | | | | | | | | |
|  | | | | | | | | | |

| Fixed Effects Parameter Estimates | | | | | | | | | | | | | | | | | |
| --- | --- | --- | --- | --- | --- | --- | --- | --- | --- | --- | --- | --- | --- | --- | --- | --- | --- |
|  | | | | | | | | **95% Confidence Interval** | | | |  | | | | | |
| **Names** | | **Effect** | | **Estimate** | | **SE** | | **Lower** | | **Upper** | | **df** | | **t** | | **p** | |
| (Intercept) |  | (Intercept) |  | 1.53 |  | 0.21 |  | 1.11 |  | 1.94 |  | 40.05 |  | 7.23 |  | < .001 |  |
| Gruppe1 |  | 2 - 1 |  | -0.42 |  | 0.46 |  | -1.31 |  | 0.48 |  | 40.41 |  | -0.92 |  | 0.365 |  |
| Time1 |  | 2 - 1 |  | -0.32 |  | 0.21 |  | -0.73 |  | 0.09 |  | 41.13 |  | -1.54 |  | 0.131 |  |
| Gewicht_t1kg |  | Gewicht_t1kg |  | -0.00 |  | 0.02 |  | -0.04 |  | 0.03 |  | 42.63 |  | -0.23 |  | 0.821 |  |
| Gruppe1 ✻ Time1 |  | 2 - 1 ✻ 2 - 1 |  | 0.72 |  | 0.42 |  | -0.10 |  | 1.54 |  | 40.99 |  | 1.73 |  | 0.092 |  |
|  | | | | | | | | | | | | | | | | | |

| Random Components | | | | | | | | | |
| --- | --- | --- | --- | --- | --- | --- | --- | --- | --- |
| **Groups** | | **Name** | | **SD** | | **Variance** | | **ICC** | |
| ID |  | (Intercept) |  | 1.20 |  | 1.45 |  | 0.61 |  |
| Residual |  |  |  | 0.97 |  | 0.94 |  |  |  |
| Anmerkung. Number of Obs: 86 , groups: ID 43 | | | | | | | | | |
|  | | | | | | | | | |

**Post Hoc Tests**

| Post Hoc Comparisons - Gruppe ✻ Time | | | | | | | | | | | | | | | | | | | |
| --- | --- | --- | --- | --- | --- | --- | --- | --- | --- | --- | --- | --- | --- | --- | --- | --- | --- | --- | --- |
| **Comparison** | | | | | | | | | |  | | | | | | | | | |
| **Gruppe** | | **Time** | |  | | **Gruppe** | | **Time** | | **Difference** | | **SE** | | **t** | | **df** | | **p_bonferroni_** | |
| 1 |  | 1 |  | - |  | 1 |  | 2 |  | 0.68 |  | 0.29 |  | 2.34 |  | 41.00 |  | 0.146 |  |
| 1 |  | 1 |  | - |  | 2 |  | 1 |  | 0.78 |  | 0.50 |  | 1.55 |  | 56.50 |  | 0.761 |  |
| 1 |  | 1 |  | - |  | 2 |  | 2 |  | 0.74 |  | 0.50 |  | 1.48 |  | 56.70 |  | 0.861 |  |
| 1 |  | 2 |  | - |  | 2 |  | 2 |  | 0.06 |  | 0.50 |  | 0.11 |  | 56.53 |  | 1.000 |  |
| 2 |  | 1 |  | - |  | 1 |  | 2 |  | -0.10 |  | 0.51 |  | -0.19 |  | 56.33 |  | 1.000 |  |
| 2 |  | 1 |  | - |  | 2 |  | 2 |  | -0.04 |  | 0.30 |  | -0.13 |  | 41.02 |  | 1.000 |  |
|  | | | | | | | | | | | | | | | | | | | |

**Estimated Marginal Means**

| Gruppe | | | | | | | | | | | |
| --- | --- | --- | --- | --- | --- | --- | --- | --- | --- | --- | --- |
|  | | | | | | | | **95% Confidence Interval** | | | |
| **Gruppe** | | **Mean** | | **SE** | | **df** | | **Lower** | | **Upper** | |
| 1 |  | 1.74 |  | 0.31 |  | 40.24 |  | 1.12 |  | 2.36 |  |
| 2 |  | 1.32 |  | 0.31 |  | 40.25 |  | 0.68 |  | 1.95 |  |
| Anmerkung. Estimated means are estimated averaging across interacting variables | | | | | | | | | | | |
|  | | | | | | | | | | | |

| Time | | | | | | | | | | | |
| --- | --- | --- | --- | --- | --- | --- | --- | --- | --- | --- | --- |
|  | | | | | | | | **95% Confidence Interval** | | | |
| **Time** | | **Mean** | | **SE** | | **df** | | **Lower** | | **Upper** | |
| 1 |  | 1.69 |  | 0.24 |  | 58.62 |  | 1.22 |  | 2.16 |  |
| 2 |  | 1.37 |  | 0.24 |  | 58.63 |  | 0.89 |  | 1.84 |  |
| Anmerkung. Estimated means are estimated averaging across interacting variables | | | | | | | | | | | |
|  | | | | | | | | | | | |

| Gruppe:Time | | | | | | | | | | | | | |
| --- | --- | --- | --- | --- | --- | --- | --- | --- | --- | --- | --- | --- | --- |
|  | | | | | | | | | | **95% Confidence Interval** | | | |
| **Gruppe** | | **Time** | | **Mean** | | **SE** | | **df** | | **Lower** | | **Upper** | |
| 1 |  | 1 |  | 2.08 |  | 0.34 |  | 57.64 |  | 1.40 |  | 2.76 |  |
| 2 |  | 1 |  | 1.30 |  | 0.35 |  | 57.39 |  | 0.60 |  | 2.00 |  |
| 1 |  | 2 |  | 1.39 |  | 0.34 |  | 57.45 |  | 0.71 |  | 2.08 |  |
| 2 |  | 2 |  | 1.34 |  | 0.35 |  | 57.61 |  | 0.64 |  | 2.03 |  |
| Anmerkung. Estimated means are estimated keeping constant other effects in the model to the mean | | | | | | | | | | | | | |
|  | | | | | | | | | | | | | |

**Effects Plots**


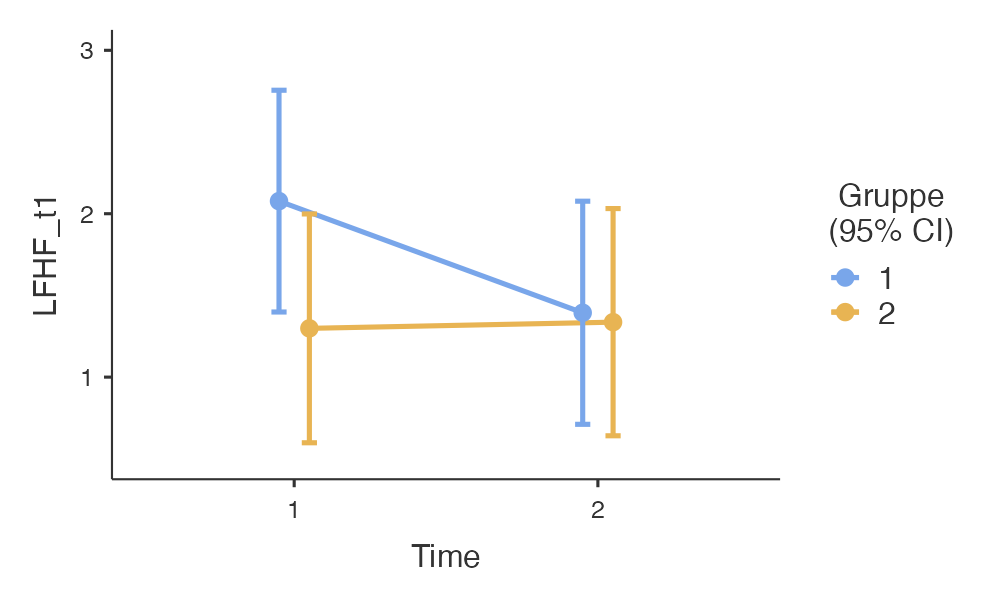


**Mixed Model LF-Power**

| Model Info | | | |
| --- | --- | --- | --- |
| **Info** | |  | |
| Estimate |  | Linear mixed model fit by REML |  |
| Call |  | LFPower_t1 ~ 1 + Gruppe + Time + Gewicht_t1kg + Gruppe:Time+( 1 \| ID ) |  |
| AIC |  | 1542.51 |  |
| BIC |  | 1496.38 |  |
| LogLikel. |  | -732.60 |  |
| R-squared Marginal |  | 0.11 |  |
| R-squared Conditional |  | 0.70 |  |
| Converged |  | yes |  |
| Optimizer |  | bobyqa |  |
|  | | | |

**Model Results**

| Fixed Effect Omnibus tests | | | | | | | | | |
| --- | --- | --- | --- | --- | --- | --- | --- | --- | --- |
|  | | **F** | | **Num df** | | **Den df** | | **p** | |
| Gruppe |  | 1.39 |  | 1 |  | 40.43 |  | 0.246 |  |
| Time |  | 3.53 |  | 1 |  | 41.09 |  | 0.067 |  |
| Gewicht_t1kg |  | 5.19 |  | 1 |  | 43.11 |  | 0.028 |  |
| Gruppe ✻ Time |  | 1.75 |  | 1 |  | 40.92 |  | 0.193 |  |
| Anmerkung. Satterthwaite method for degrees of freedom | | | | | | | | | |
|  | | | | | | | | | |

| Fixed Effects Parameter Estimates | | | | | | | | | | | | | | | | | |
| --- | --- | --- | --- | --- | --- | --- | --- | --- | --- | --- | --- | --- | --- | --- | --- | --- | --- |
|  | | | | | | | | **95% Confidence Interval** | | | |  | | | | | |
| **Names** | | **Effect** | | **Estimate** | | **SE** | | **Lower** | | **Upper** | | **df** | | **t** | | **p** | |
| (Intercept) |  | (Intercept) |  | 1907.79 |  | 289.70 |  | 1339.99 |  | 2475.59 |  | 40.00 |  | 6.59 |  | < .001 |  |
| Gruppe1 |  | 2 - 1 |  | 737.87 |  | 626.36 |  | -489.78 |  | 1965.52 |  | 40.43 |  | 1.18 |  | 0.246 |  |
| Time1 |  | 2 - 1 |  | 491.50 |  | 261.66 |  | -21.35 |  | 1004.36 |  | 41.09 |  | 1.88 |  | 0.067 |  |
| Gewicht_t1kg |  | Gewicht_t1kg |  | -60.89 |  | 26.74 |  | -113.29 |  | -8.49 |  | 43.11 |  | -2.28 |  | 0.028 |  |
| Gruppe1 ✻ Time1 |  | 2 - 1 ✻ 2 - 1 |  | 692.50 |  | 522.78 |  | -332.13 |  | 1717.12 |  | 40.92 |  | 1.32 |  | 0.193 |  |
|  | | | | | | | | | | | | | | | | | |

| Random Components | | | | | | | | | |
| --- | --- | --- | --- | --- | --- | --- | --- | --- | --- |
| **Groups** | | **Name** | | **SD** | | **Variance** | | **ICC** | |
| ID |  | (Intercept) |  | 1694.83 |  | 2872447.92 |  | 0.66 |  |
| Residual |  |  |  | 1211.68 |  | 1468160.53 |  |  |  |
| Anmerkung. Number of Obs: 86 , groups: ID 43 | | | | | | | | | |
|  | | | | | | | | | |

**Post Hoc Tests**

| Post Hoc Comparisons - Gruppe ✻ Time | | | | | | | | | | | | | | | | | | | |
| --- | --- | --- | --- | --- | --- | --- | --- | --- | --- | --- | --- | --- | --- | --- | --- | --- | --- | --- | --- |
| **Comparison** | | | | | | | | | |  | | | | | | | | | |
| **Gruppe** | | **Time** | |  | | **Gruppe** | | **Time** | | **Difference** | | **SE** | | **t** | | **df** | | **p_bonferroni_** | |
| 1 |  | 1 |  | - |  | 1 |  | 2 |  | -145.25 |  | 365.50 |  | -0.40 |  | 41.00 |  | 1.000 |  |
| 1 |  | 1 |  | - |  | 2 |  | 1 |  | -391.62 |  | 679.35 |  | -0.58 |  | 54.03 |  | 1.000 |  |
| 1 |  | 1 |  | - |  | 2 |  | 2 |  | -1229.37 |  | 674.86 |  | -1.82 |  | 54.19 |  | 0.444 |  |
| 1 |  | 2 |  | - |  | 2 |  | 2 |  | -1084.12 |  | 678.68 |  | -1.60 |  | 54.06 |  | 0.696 |  |
| 2 |  | 1 |  | - |  | 1 |  | 2 |  | 246.36 |  | 683.36 |  | 0.36 |  | 53.90 |  | 1.000 |  |
| 2 |  | 1 |  | - |  | 2 |  | 2 |  | -837.75 |  | 374.16 |  | -2.24 |  | 41.02 |  | 0.184 |  |
|  | | | | | | | | | | | | | | | | | | | |

**Estimated Marginal Means**

| Gruppe | | | | | | | | | | | |
| --- | --- | --- | --- | --- | --- | --- | --- | --- | --- | --- | --- |
|  | | | | | | | | **95% Confidence Interval** | | | |
| **Gruppe** | | **Mean** | | **SE** | | **df** | | **Lower** | | **Upper** | |
| 1 |  | 1538.85 |  | 421.24 |  | 40.23 |  | 687.63 |  | 2390.07 |  |
| 2 |  | 2276.72 |  | 431.94 |  | 40.24 |  | 1403.90 |  | 3149.54 |  |
| Anmerkung. Estimated means are estimated averaging across interacting variables | | | | | | | | | | | |
|  | | | | | | | | | | | |

| Time | | | | | | | | | | | |
| --- | --- | --- | --- | --- | --- | --- | --- | --- | --- | --- | --- |
|  | | | | | | | | **95% Confidence Interval** | | | |
| **Time** | | **Mean** | | **SE** | | **df** | | **Lower** | | **Upper** | |
| 1 |  | 1662.03 |  | 317.92 |  | 55.69 |  | 1025.08 |  | 2298.99 |  |
| 2 |  | 2153.54 |  | 317.82 |  | 55.70 |  | 1516.79 |  | 2790.28 |  |
| Anmerkung. Estimated means are estimated averaging across interacting variables | | | | | | | | | | | |
|  | | | | | | | | | | | |

| Gruppe:Time | | | | | | | | | | | | | |
| --- | --- | --- | --- | --- | --- | --- | --- | --- | --- | --- | --- | --- | --- |
|  | | | | | | | | | | **95% Confidence Interval** | | | |
| **Gruppe** | | **Time** | | **Mean** | | **SE** | | **df** | | **Lower** | | **Upper** | |
| 1 |  | 1 |  | 1466.23 |  | 457.77 |  | 54.91 |  | 548.79 |  | 2383.66 |  |
| 2 |  | 1 |  | 1857.84 |  | 472.39 |  | 54.70 |  | 911.04 |  | 2804.65 |  |
| 1 |  | 2 |  | 1611.48 |  | 460.58 |  | 54.75 |  | 688.37 |  | 2534.59 |  |
| 2 |  | 2 |  | 2695.59 |  | 469.03 |  | 54.88 |  | 1755.60 |  | 3635.59 |  |
| Anmerkung. Estimated means are estimated keeping constant other effects in the model to the mean | | | | | | | | | | | | | |
|  | | | | | | | | | | | | | |

**Effects Plots**


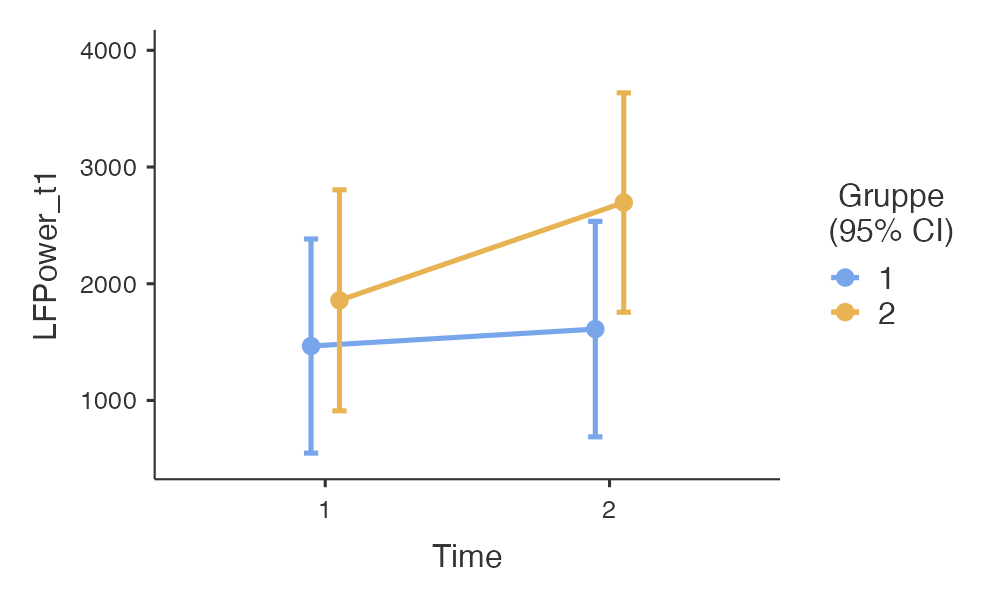


**Mixed Model HF-Power**

| Model Info | | | |
| --- | --- | --- | --- |
| **Info** | |  | |
| Estimate |  | Linear mixed model fit by REML |  |
| Call |  | HFPower_t1 ~ 1 + Gruppe + Time + Gewicht_t1kg + Gruppe:Time+( 1 \| ID ) |  |
| AIC |  | 1531.29 |  |
| BIC |  | 1485.27 |  |
| LogLikel. |  | -727.04 |  |
| R-squared Marginal |  | 0.12 |  |
| R-squared Conditional |  | 0.90 |  |
| Converged |  | yes |  |
| Optimizer |  | bobyqa |  |
|  | | | |

**Model Results**

| Fixed Effect Omnibus tests | | | | | | | | | |
| --- | --- | --- | --- | --- | --- | --- | --- | --- | --- |
|  | | **F** | | **Num df** | | **Den df** | | **p** | |
| Gruppe |  | 4.13 |  | 1 |  | 41.47 |  | 0.049 |  |
| Time |  | 10.80 |  | 1 |  | 41.42 |  | 0.002 |  |
| Gewicht_t1kg |  | 4.03 |  | 1 |  | 50.84 |  | 0.050 |  |
| Gruppe ✻ Time |  | 1.01 |  | 1 |  | 40.94 |  | 0.322 |  |
| Anmerkung. Satterthwaite method for degrees of freedom | | | | | | | | | |
|  | | | | | | | | | |

| Fixed Effects Parameter Estimates | | | | | | | | | | | | | | | | | |
| --- | --- | --- | --- | --- | --- | --- | --- | --- | --- | --- | --- | --- | --- | --- | --- | --- | --- |
|  | | | | | | | | **95% Confidence Interval** | | | |  | | | | | |
| **Names** | | **Effect** | | **Estimate** | | **SE** | | **Lower** | | **Upper** | | **df** | | **t** | | **p** | |
| (Intercept) |  | (Intercept) |  | 1920.96 |  | 370.58 |  | 1194.65 |  | 2647.28 |  | 40.17 |  | 5.18 |  | < .001 |  |
| Gruppe1 |  | 2 - 1 |  | 1617.48 |  | 796.31 |  | 56.74 |  | 3178.21 |  | 41.47 |  | 2.03 |  | 0.049 |  |
| Time1 |  | 2 - 1 |  | 591.27 |  | 179.94 |  | 238.59 |  | 943.94 |  | 41.42 |  | 3.29 |  | 0.002 |  |
| Gewicht_t1kg |  | Gewicht_t1kg |  | -65.67 |  | 32.72 |  | -129.79 |  | -1.55 |  | 50.84 |  | -2.01 |  | 0.050 |  |
| Gruppe1 ✻ Time1 |  | 2 - 1 ✻ 2 - 1 |  | -359.59 |  | 358.68 |  | -1062.59 |  | 343.41 |  | 40.94 |  | -1.00 |  | 0.322 |  |
|  | | | | | | | | | | | | | | | | | |

| Random Components | | | | | | | | | |
| --- | --- | --- | --- | --- | --- | --- | --- | --- | --- |
| **Groups** | | **Name** | | **SD** | | **Variance** | | **ICC** | |
| ID |  | (Intercept) |  | 2357.08 |  | 5555833.51 |  | 0.89 |  |
| Residual |  |  |  | 831.32 |  | 691094.09 |  |  |  |
| Anmerkung. Number of Obs: 86 , groups: ID 43 | | | | | | | | | |
|  | | | | | | | | | |

**Post Hoc Tests**

| Post Hoc Comparisons - Gruppe ✻ Time | | | | | | | | | | | | | | | | | | | |
| --- | --- | --- | --- | --- | --- | --- | --- | --- | --- | --- | --- | --- | --- | --- | --- | --- | --- | --- | --- |
| **Comparison** | | | | | | | | | |  | | | | | | | | | |
| **Gruppe** | | **Time** | |  | | **Gruppe** | | **Time** | | **Difference** | | **SE** | | **t** | | **df** | | **p_bonferroni_** | |
| 1 |  | 1 |  | - |  | 1 |  | 2 |  | -771.06 |  | 251.03 |  | -3.07 |  | 40.99 |  | 0.023 |  |
| 1 |  | 1 |  | - |  | 2 |  | 1 |  | -1797.27 |  | 817.76 |  | -2.20 |  | 45.43 |  | 0.199 |  |
| 1 |  | 1 |  | - |  | 2 |  | 2 |  | -2208.74 |  | 812.10 |  | -2.72 |  | 45.37 |  | 0.055 |  |
| 1 |  | 2 |  | - |  | 2 |  | 2 |  | -1437.68 |  | 816.91 |  | -1.76 |  | 45.42 |  | 0.511 |  |
| 2 |  | 1 |  | - |  | 1 |  | 2 |  | 1026.21 |  | 822.81 |  | 1.25 |  | 45.50 |  | 1.000 |  |
| 2 |  | 1 |  | - |  | 2 |  | 2 |  | -411.47 |  | 257.05 |  | -1.60 |  | 41.05 |  | 0.703 |  |
|  | | | | | | | | | | | | | | | | | | | |

**Estimated Marginal Means**

| Gruppe | | | | | | | | | | | |
| --- | --- | --- | --- | --- | --- | --- | --- | --- | --- | --- | --- |
|  | | | | | | | | **95% Confidence Interval** | | | |
| **Gruppe** | | **Mean** | | **SE** | | **df** | | **Lower** | | **Upper** | |
| 1 |  | 1112.22 |  | 537.10 |  | 40.85 |  | 27.40 |  | 2197.05 |  |
| 2 |  | 2729.70 |  | 550.66 |  | 40.88 |  | 1617.52 |  | 3841.88 |  |
| Anmerkung. Estimated means are estimated averaging across interacting variables | | | | | | | | | | | |
|  | | | | | | | | | | | |

| Time | | | | | | | | | | | |
| --- | --- | --- | --- | --- | --- | --- | --- | --- | --- | --- | --- |
|  | | | | | | | | **95% Confidence Interval** | | | |
| **Time** | | **Mean** | | **SE** | | **df** | | **Lower** | | **Upper** | |
| 1 |  | 1625.33 |  | 381.41 |  | 44.86 |  | 857.07 |  | 2393.59 |  |
| 2 |  | 2216.60 |  | 381.28 |  | 44.86 |  | 1448.60 |  | 2984.59 |  |
| Anmerkung. Estimated means are estimated averaging across interacting variables | | | | | | | | | | | |
|  | | | | | | | | | | | |

| Gruppe:Time | | | | | | | | | | | | | |
| --- | --- | --- | --- | --- | --- | --- | --- | --- | --- | --- | --- | --- | --- |
|  | | | | | | | | | | **95% Confidence Interval** | | | |
| **Gruppe** | | **Time** | | **Mean** | | **SE** | | **df** | | **Lower** | | **Upper** | |
| 1 |  | 1 |  | 726.69 |  | 549.82 |  | 45.20 |  | -380.57 |  | 1833.96 |  |
| 2 |  | 1 |  | 2523.97 |  | 567.55 |  | 45.29 |  | 1381.06 |  | 3666.87 |  |
| 1 |  | 2 |  | 1497.76 |  | 553.32 |  | 45.27 |  | 383.49 |  | 2612.02 |  |
| 2 |  | 2 |  | 2935.44 |  | 563.36 |  | 45.21 |  | 1800.91 |  | 4069.96 |  |
| Anmerkung. Estimated means are estimated keeping constant other effects in the model to the mean | | | | | | | | | | | | | |
|  | | | | | | | | | | | | | |

**Effects Plots**


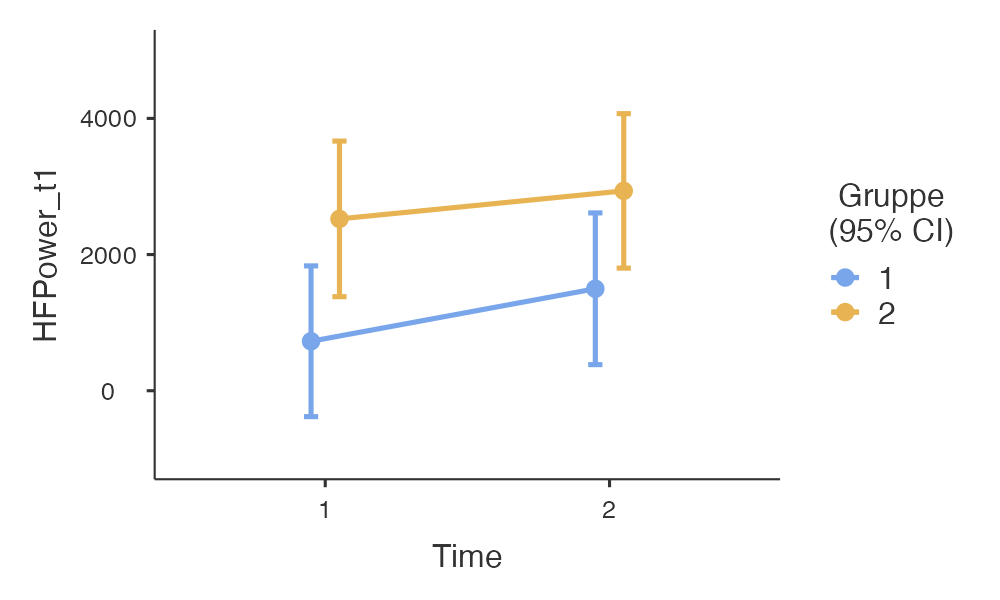


**Mixed Model SBP**

| Model Info | | | |
| --- | --- | --- | --- |
| **Info** | |  | |
| Estimate |  | Linear mixed model fit by REML |  |
| Call |  | pSBPmean_t1 ~ 1 + Gruppe + Time + Gewicht_t1kg + Gruppe:Time+( 1 \| ID ) |  |
| AIC |  | 586.73 |  |
| BIC |  | 595.83 |  |
| LogLikel. |  | -282.32 |  |
| R-squared Marginal |  | 0.18 |  |
| R-squared Conditional |  | 0.86 |  |
| Converged |  | yes |  |
| Optimizer |  | bobyqa |  |
|  | | | |

**Model Results**

| Fixed Effect Omnibus tests | | | | | | | | | |
| --- | --- | --- | --- | --- | --- | --- | --- | --- | --- |
|  | | **F** | | **Num df** | | **Den df** | | **p** | |
| Gruppe |  | 0.26 |  | 1 |  | 40.98 |  | 0.610 |  |
| Time |  | 30.81 |  | 1 |  | 41.30 |  | < .001 |  |
| Gewicht_t1kg |  | 6.83 |  | 1 |  | 46.68 |  | 0.012 |  |
| Gruppe ✻ Time |  | 7.00 |  | 1 |  | 40.97 |  | 0.012 |  |
| Anmerkung. Satterthwaite method for degrees of freedom | | | | | | | | | |
|  | | | | | | | | | |

| Fixed Effects Parameter Estimates | | | | | | | | | | | | | | | | | |
| --- | --- | --- | --- | --- | --- | --- | --- | --- | --- | --- | --- | --- | --- | --- | --- | --- | --- |
|  | | | | | | | | **95% Confidence Interval** | | | |  | | | | | |
| **Names** | | **Effect** | | **Estimate** | | **SE** | | **Lower** | | **Upper** | | **df** | | **t** | | **p** | |
| (Intercept) |  | (Intercept) |  | 117.07 |  | 1.35 |  | 114.43 |  | 119.71 |  | 40.13 |  | 86.78 |  | < .001 |  |
| Gruppe1 |  | 2 - 1 |  | -1.50 |  | 2.91 |  | -7.20 |  | 4.20 |  | 40.98 |  | -0.51 |  | 0.610 |  |
| Time1 |  | 2 - 1 |  | -4.65 |  | 0.84 |  | -6.30 |  | -3.01 |  | 41.30 |  | -5.55 |  | < .001 |  |
| Gewicht_t1kg |  | Gewicht_t1kg |  | 0.32 |  | 0.12 |  | 0.08 |  | 0.56 |  | 46.68 |  | 2.61 |  | 0.012 |  |
| Gruppe1 ✻ Time1 |  | 2 - 1 ✻ 2 - 1 |  | 4.43 |  | 1.67 |  | 1.15 |  | 7.71 |  | 40.97 |  | 2.65 |  | 0.012 |  |
|  | | | | | | | | | | | | | | | | | |

| Random Components | | | | | | | | | |
| --- | --- | --- | --- | --- | --- | --- | --- | --- | --- |
| **Groups** | | **Name** | | **SD** | | **Variance** | | **ICC** | |
| ID |  | (Intercept) |  | 8.41 |  | 70.68 |  | 0.82 |  |
| Residual |  |  |  | 3.88 |  | 15.04 |  |  |  |
| Anmerkung. Number of Obs: 86 , groups: ID 43 | | | | | | | | | |
|  | | | | | | | | | |

**Post Hoc Tests**

| Post Hoc Comparisons - Gruppe ✻ Time | | | | | | | | | | | | | | | | | | | |
| --- | --- | --- | --- | --- | --- | --- | --- | --- | --- | --- | --- | --- | --- | --- | --- | --- | --- | --- | --- |
| **Comparison** | | | | | | | | | |  | | | | | | | | | |
| **Gruppe** | | **Time** | |  | | **Gruppe** | | **Time** | | **Difference** | | **SE** | | **t** | | **df** | | **p_bonferroni_** | |
| 1 |  | 1 |  | - |  | 1 |  | 2 |  | 6.87 |  | 1.17 |  | 5.87 |  | 40.99 |  | < .001 |  |
| 1 |  | 1 |  | - |  | 2 |  | 1 |  | 3.71 |  | 3.03 |  | 1.22 |  | 47.51 |  | 1.000 |  |
| 1 |  | 1 |  | - |  | 2 |  | 2 |  | 6.15 |  | 3.01 |  | 2.04 |  | 47.53 |  | 0.279 |  |
| 1 |  | 2 |  | - |  | 2 |  | 2 |  | -0.72 |  | 3.03 |  | -0.24 |  | 47.51 |  | 1.000 |  |
| 2 |  | 1 |  | - |  | 1 |  | 2 |  | 3.16 |  | 3.05 |  | 1.04 |  | 47.50 |  | 1.000 |  |
| 2 |  | 1 |  | - |  | 2 |  | 2 |  | 2.44 |  | 1.20 |  | 2.04 |  | 41.04 |  | 0.289 |  |
|  | | | | | | | | | | | | | | | | | | | |

**Estimated Marginal Means**

| Gruppe | | | | | | | | | | | |
| --- | --- | --- | --- | --- | --- | --- | --- | --- | --- | --- | --- |
|  | | | | | | | | **95% Confidence Interval** | | | |
| **Gruppe** | | **Mean** | | **SE** | | **df** | | **Lower** | | **Upper** | |
| 1 |  | 117.82 |  | 1.96 |  | 40.57 |  | 113.86 |  | 121.77 |  |
| 2 |  | 116.32 |  | 2.01 |  | 40.59 |  | 112.27 |  | 120.38 |  |
| Anmerkung. Estimated means are estimated averaging across interacting variables | | | | | | | | | | | |
|  | | | | | | | | | | | |

| Time | | | | | | | | | | | |
| --- | --- | --- | --- | --- | --- | --- | --- | --- | --- | --- | --- |
|  | | | | | | | | **95% Confidence Interval** | | | |
| **Time** | | **Mean** | | **SE** | | **df** | | **Lower** | | **Upper** | |
| 1 |  | 119.40 |  | 1.41 |  | 47.77 |  | 116.56 |  | 122.24 |  |
| 2 |  | 114.74 |  | 1.41 |  | 47.77 |  | 111.90 |  | 117.58 |  |
| Anmerkung. Estimated means are estimated averaging across interacting variables | | | | | | | | | | | |
|  | | | | | | | | | | | |

| Gruppe:Time | | | | | | | | | | | | | |
| --- | --- | --- | --- | --- | --- | --- | --- | --- | --- | --- | --- | --- | --- |
|  | | | | | | | | | | **95% Confidence Interval** | | | |
| **Gruppe** | | **Time** | | **Mean** | | **SE** | | **df** | | **Lower** | | **Upper** | |
| 1 |  | 1 |  | 121.25 |  | 2.04 |  | 47.70 |  | 117.15 |  | 125.35 |  |
| 2 |  | 1 |  | 117.54 |  | 2.10 |  | 47.69 |  | 113.31 |  | 121.77 |  |
| 1 |  | 2 |  | 114.38 |  | 2.05 |  | 47.69 |  | 110.26 |  | 118.51 |  |
| 2 |  | 2 |  | 115.10 |  | 2.09 |  | 47.70 |  | 110.90 |  | 119.30 |  |
| Anmerkung. Estimated means are estimated keeping constant other effects in the model to the mean | | | | | | | | | | | | | |
|  | | | | | | | | | | | | | |

**Effects Plots**


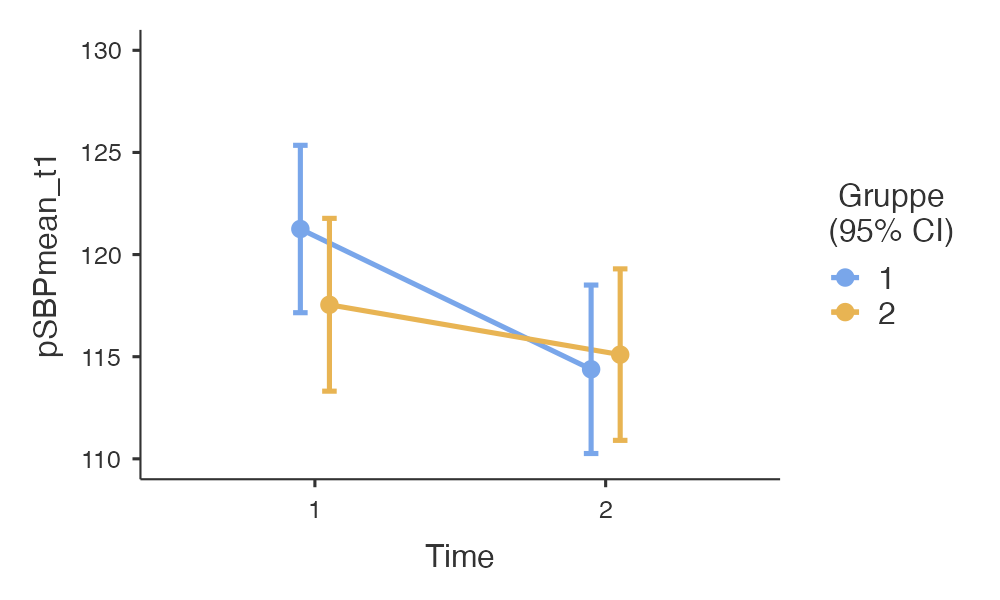


**Mixed Model DBP**

| Model Info | | | |
| --- | --- | --- | --- |
| **Info** | |  | |
| Estimate |  | Linear mixed model fit by REML |  |
| Call |  | pDBPmean_t1 ~ 1 + Gruppe + Time + Gewicht_t1kg + Gruppe:Time+( 1 \| ID ) |  |
| AIC |  | 577.72 |  |
| BIC |  | 587.43 |  |
| LogLikel. |  | -278.12 |  |
| R-squared Marginal |  | 0.10 |  |
| R-squared Conditional |  | 0.81 |  |
| Converged |  | yes |  |
| Optimizer |  | bobyqa |  |
|  | | | |

**Model Results**

| Fixed Effect Omnibus tests | | | | | | | | | |
| --- | --- | --- | --- | --- | --- | --- | --- | --- | --- |
|  | | **F** | | **Num df** | | **Den df** | | **p** | |
| Gruppe |  | 0.29 |  | 1 |  | 40.84 |  | 0.595 |  |
| Time |  | 4.53 |  | 1 |  | 41.27 |  | 0.039 |  |
| Gewicht_t1kg |  | 4.52 |  | 1 |  | 45.49 |  | 0.039 |  |
| Gruppe ✻ Time |  | 2.70 |  | 1 |  | 41.00 |  | 0.108 |  |
| Anmerkung. Satterthwaite method for degrees of freedom | | | | | | | | | |
|  | | | | | | | | | |

| Fixed Effects Parameter Estimates | | | | | | | | | | | | | | | | | |
| --- | --- | --- | --- | --- | --- | --- | --- | --- | --- | --- | --- | --- | --- | --- | --- | --- | --- |
|  | | | | | | | | **95% Confidence Interval** | | | |  | | | | | |
| **Names** | | **Effect** | | **Estimate** | | **SE** | | **Lower** | | **Upper** | | **df** | | **t** | | **p** | |
| (Intercept) |  | (Intercept) |  | 73.06 |  | 1.22 |  | 70.67 |  | 75.44 |  | 40.12 |  | 60.03 |  | < .001 |  |
| Gruppe1 |  | 2 - 1 |  | -1.41 |  | 2.63 |  | -6.56 |  | 3.74 |  | 40.84 |  | -0.54 |  | 0.595 |  |
| Time1 |  | 2 - 1 |  | -1.78 |  | 0.84 |  | -3.42 |  | -0.14 |  | 41.27 |  | -2.13 |  | 0.039 |  |
| Gewicht_t1kg |  | Gewicht_t1kg |  | 0.24 |  | 0.11 |  | 0.02 |  | 0.45 |  | 45.49 |  | 2.13 |  | 0.039 |  |
| Gruppe1 ✻ Time1 |  | 2 - 1 ✻ 2 - 1 |  | 2.74 |  | 1.67 |  | -0.53 |  | 6.02 |  | 41.00 |  | 1.64 |  | 0.108 |  |
|  | | | | | | | | | | | | | | | | | |

| Random Components | | | | | | | | | |
| --- | --- | --- | --- | --- | --- | --- | --- | --- | --- |
| **Groups** | | **Name** | | **SD** | | **Variance** | | **ICC** | |
| ID |  | (Intercept) |  | 7.49 |  | 56.16 |  | 0.79 |  |
| Residual |  |  |  | 3.87 |  | 14.99 |  |  |  |
| Anmerkung. Number of Obs: 86 , groups: ID 43 | | | | | | | | | |
|  | | | | | | | | | |

**Post Hoc Tests**

| Post Hoc Comparisons - Gruppe ✻ Time | | | | | | | | | | | | | | | | | | | |
| --- | --- | --- | --- | --- | --- | --- | --- | --- | --- | --- | --- | --- | --- | --- | --- | --- | --- | --- | --- |
| **Comparison** | | | | | | | | | |  | | | | | | | | | |
| **Gruppe** | | **Time** | |  | | **Gruppe** | | **Time** | | **Difference** | | **SE** | | **t** | | **df** | | **p_bonferroni_** | |
| 1 |  | 1 |  | - |  | 1 |  | 2 |  | 3.15 |  | 1.17 |  | 2.70 |  | 41.00 |  | 0.060 |  |
| 1 |  | 1 |  | - |  | 2 |  | 1 |  | 2.78 |  | 2.76 |  | 1.01 |  | 48.80 |  | 1.000 |  |
| 1 |  | 1 |  | - |  | 2 |  | 2 |  | 3.19 |  | 2.74 |  | 1.16 |  | 48.85 |  | 1.000 |  |
| 1 |  | 2 |  | - |  | 2 |  | 2 |  | 0.04 |  | 2.76 |  | 0.01 |  | 48.81 |  | 1.000 |  |
| 2 |  | 1 |  | - |  | 1 |  | 2 |  | 0.37 |  | 2.78 |  | 0.13 |  | 48.76 |  | 1.000 |  |
| 2 |  | 1 |  | - |  | 2 |  | 2 |  | 0.41 |  | 1.20 |  | 0.34 |  | 41.03 |  | 1.000 |  |
|  | | | | | | | | | | | | | | | | | | | |

**Estimated Marginal Means**

| Gruppe | | | | | | | | | | | |
| --- | --- | --- | --- | --- | --- | --- | --- | --- | --- | --- | --- |
|  | | | | | | | | **95% Confidence Interval** | | | |
| **Gruppe** | | **Mean** | | **SE** | | **df** | | **Lower** | | **Upper** | |
| 1 |  | 73.76 |  | 1.77 |  | 40.50 |  | 70.19 |  | 77.33 |  |
| 2 |  | 72.36 |  | 1.81 |  | 40.51 |  | 68.69 |  | 76.02 |  |
| Anmerkung. Estimated means are estimated averaging across interacting variables | | | | | | | | | | | |
|  | | | | | | | | | | | |

| Time | | | | | | | | | | | |
| --- | --- | --- | --- | --- | --- | --- | --- | --- | --- | --- | --- |
|  | | | | | | | | **95% Confidence Interval** | | | |
| **Time** | | **Mean** | | **SE** | | **df** | | **Lower** | | **Upper** | |
| 1 |  | 73.95 |  | 1.29 |  | 49.43 |  | 71.36 |  | 76.54 |  |
| 2 |  | 72.17 |  | 1.29 |  | 49.44 |  | 69.58 |  | 74.75 |  |
| Anmerkung. Estimated means are estimated averaging across interacting variables | | | | | | | | | | | |
|  | | | | | | | | | | | |

| Gruppe:Time | | | | | | | | | | | | | |
| --- | --- | --- | --- | --- | --- | --- | --- | --- | --- | --- | --- | --- | --- |
|  | | | | | | | | | | **95% Confidence Interval** | | | |
| **Gruppe** | | **Time** | | **Mean** | | **SE** | | **df** | | **Lower** | | **Upper** | |
| 1 |  | 1 |  | 75.34 |  | 1.86 |  | 49.19 |  | 71.61 |  | 79.07 |  |
| 2 |  | 1 |  | 72.56 |  | 1.92 |  | 49.12 |  | 68.71 |  | 76.41 |  |
| 1 |  | 2 |  | 72.19 |  | 1.87 |  | 49.14 |  | 68.43 |  | 75.94 |  |
| 2 |  | 2 |  | 72.15 |  | 1.90 |  | 49.18 |  | 68.33 |  | 75.97 |  |
| Anmerkung. Estimated means are estimated keeping constant other effects in the model to the mean | | | | | | | | | | | | | |
|  | | | | | | | | | | | | | |

**Effects Plots**


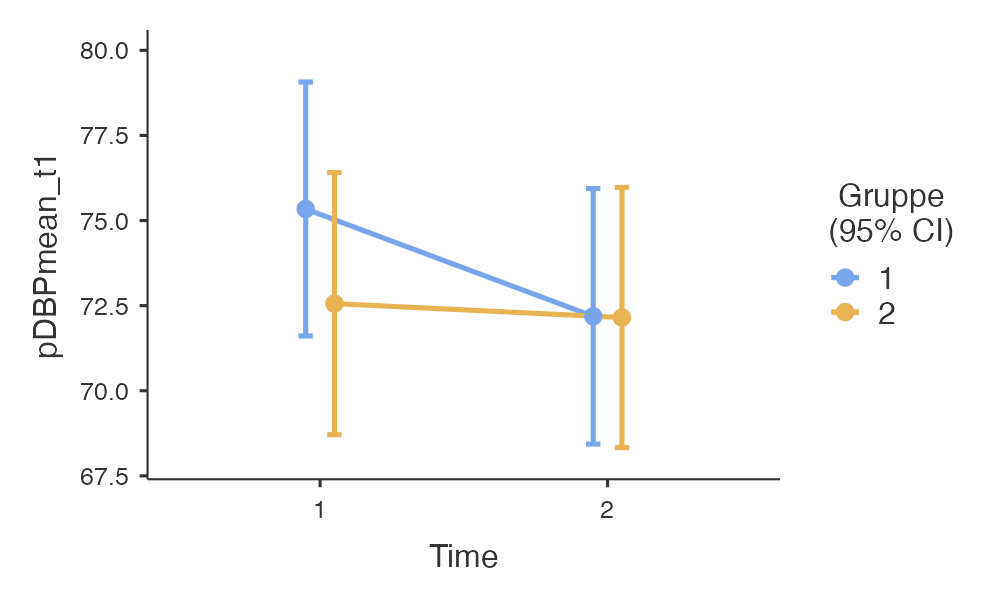


**Mixed Model VO2peak**

| Model Info | | | |
| --- | --- | --- | --- |
| **Info** | |  | |
| Estimate |  | Linear mixed model fit by REML |  |
| Call |  | VO2kgmax_t1 ~ 1 + Gruppe + Time + Gewicht_t1kg + Gruppe:Time+( 1 \| ID ) |  |
| AIC |  | 512.86 |  |
| BIC |  | 525.68 |  |
| LogLikel. |  | -247.25 |  |
| R-squared Marginal |  | 0.03 |  |
| R-squared Conditional |  | 0.97 |  |
| Converged |  | yes |  |
| Optimizer |  | bobyqa |  |
|  | | | |

**Model Results**

| Fixed Effect Omnibus tests | | | | | | | | | |
| --- | --- | --- | --- | --- | --- | --- | --- | --- | --- |
|  | | **F** | | **Num df** | | **Den df** | | **p** | |
| Gruppe |  | 0.05 |  | 1 |  | 42.86 |  | 0.816 |  |
| Time |  | 40.24 |  | 1 |  | 41.35 |  | < .001 |  |
| Gewicht_t1kg |  | 0.04 |  | 1 |  | 71.62 |  | 0.848 |  |
| Gruppe ✻ Time |  | 18.11 |  | 1 |  | 40.36 |  | < .001 |  |
| Anmerkung. Satterthwaite method for degrees of freedom | | | | | | | | | |
|  | | | | | | | | | |

| Fixed Effects Parameter Estimates | | | | | | | | | | | | | | | | | |
| --- | --- | --- | --- | --- | --- | --- | --- | --- | --- | --- | --- | --- | --- | --- | --- | --- | --- |
|  | | | | | | | | **95% Confidence Interval** | | | |  | | | | | |
| **Names** | | **Effect** | | **Estimate** | | **SE** | | **Lower** | | **Upper** | | **df** | | **t** | | **p** | |
| (Intercept) |  | (Intercept) |  | 36.66 |  | 1.36 |  | 33.99 |  | 39.33 |  | 40.00 |  | 26.92 |  | < .001 |  |
| Gruppe1 |  | 2 - 1 |  | -0.67 |  | 2.88 |  | -6.32 |  | 4.97 |  | 42.86 |  | -0.23 |  | 0.816 |  |
| Time1 |  | 2 - 1 |  | 2.25 |  | 0.35 |  | 1.55 |  | 2.94 |  | 41.35 |  | 6.34 |  | < .001 |  |
| Gewicht_t1kg |  | Gewicht_t1kg |  | -0.02 |  | 0.11 |  | -0.23 |  | 0.19 |  | 71.62 |  | -0.19 |  | 0.848 |  |
| Gruppe1 ✻ Time1 |  | 2 - 1 ✻ 2 - 1 |  | -2.99 |  | 0.70 |  | -4.36 |  | -1.61 |  | 40.36 |  | -4.26 |  | < .001 |  |
|  | | | | | | | | | | | | | | | | | |

| Random Components | | | | | | | | | |
| --- | --- | --- | --- | --- | --- | --- | --- | --- | --- |
| **Groups** | | **Name** | | **SD** | | **Variance** | | **ICC** | |
| ID |  | (Intercept) |  | 8.85 |  | 78.37 |  | 0.97 |  |
| Residual |  |  |  | 1.63 |  | 2.65 |  |  |  |
| Anmerkung. Number of Obs: 86 , groups: ID 43 | | | | | | | | | |
|  | | | | | | | | | |

**Post Hoc Tests**

| Post Hoc Comparisons - Gruppe ✻ Time | | | | | | | | | | | | | | | | | | | |
| --- | --- | --- | --- | --- | --- | --- | --- | --- | --- | --- | --- | --- | --- | --- | --- | --- | --- | --- | --- |
| **Comparison** | | | | | | | | | |  | | | | | | | | | |
| **Gruppe** | | **Time** | |  | | **Gruppe** | | **Time** | | **Difference** | | **SE** | | **t** | | **df** | | **p_bonferroni_** | |
| 1 |  | 1 |  | - |  | 1 |  | 2 |  | -3.74 |  | 0.49 |  | -7.59 |  | 40.89 |  | < .001 |  |
| 1 |  | 1 |  | - |  | 2 |  | 1 |  | -0.82 |  | 2.91 |  | -0.28 |  | 44.22 |  | 1.000 |  |
| 1 |  | 1 |  | - |  | 2 |  | 2 |  | -1.57 |  | 2.89 |  | -0.54 |  | 43.94 |  | 1.000 |  |
| 1 |  | 2 |  | - |  | 2 |  | 2 |  | 2.17 |  | 2.91 |  | 0.75 |  | 44.18 |  | 1.000 |  |
| 2 |  | 1 |  | - |  | 1 |  | 2 |  | -2.92 |  | 2.93 |  | -1.00 |  | 44.46 |  | 1.000 |  |
| 2 |  | 1 |  | - |  | 2 |  | 2 |  | -0.75 |  | 0.50 |  | -1.49 |  | 41.03 |  | 0.861 |  |
|  | | | | | | | | | | | | | | | | | | | |

**Estimated Marginal Means**

| Gruppe | | | | | | | | | | | |
| --- | --- | --- | --- | --- | --- | --- | --- | --- | --- | --- | --- |
|  | | | | | | | | **95% Confidence Interval** | | | |
| **Gruppe** | | **Mean** | | **SE** | | **df** | | **Lower** | | **Upper** | |
| 1 |  | 37.00 |  | 1.96 |  | 41.45 |  | 33.05 |  | 40.95 |  |
| 2 |  | 36.33 |  | 2.01 |  | 41.52 |  | 32.28 |  | 40.38 |  |
| Anmerkung. Estimated means are estimated averaging across interacting variables | | | | | | | | | | | |
|  | | | | | | | | | | | |

| Time | | | | | | | | | | | |
| --- | --- | --- | --- | --- | --- | --- | --- | --- | --- | --- | --- |
|  | | | | | | | | **95% Confidence Interval** | | | |
| **Time** | | **Mean** | | **SE** | | **df** | | **Lower** | | **Upper** | |
| 1 |  | 35.54 |  | 1.37 |  | 41.35 |  | 32.77 |  | 38.31 |  |
| 2 |  | 37.79 |  | 1.37 |  | 41.34 |  | 35.01 |  | 40.56 |  |
| Anmerkung. Estimated means are estimated averaging across interacting variables | | | | | | | | | | | |
|  | | | | | | | | | | | |

| Gruppe:Time | | | | | | | | | | | | | |
| --- | --- | --- | --- | --- | --- | --- | --- | --- | --- | --- | --- | --- | --- |
|  | | | | | | | | | | **95% Confidence Interval** | | | |
| **Gruppe** | | **Time** | | **Mean** | | **SE** | | **df** | | **Lower** | | **Upper** | |
| 1 |  | 1 |  | 35.13 |  | 1.97 |  | 42.61 |  | 31.16 |  | 39.10 |  |
| 2 |  | 1 |  | 35.95 |  | 2.03 |  | 42.95 |  | 31.86 |  | 40.04 |  |
| 1 |  | 2 |  | 38.87 |  | 1.98 |  | 42.87 |  | 34.88 |  | 42.86 |  |
| 2 |  | 2 |  | 36.70 |  | 2.02 |  | 42.65 |  | 32.64 |  | 40.77 |  |
| Anmerkung. Estimated means are estimated keeping constant other effects in the model to the mean | | | | | | | | | | | | | |
|  | | | | | | | | | | | | | |

**Effects Plots**


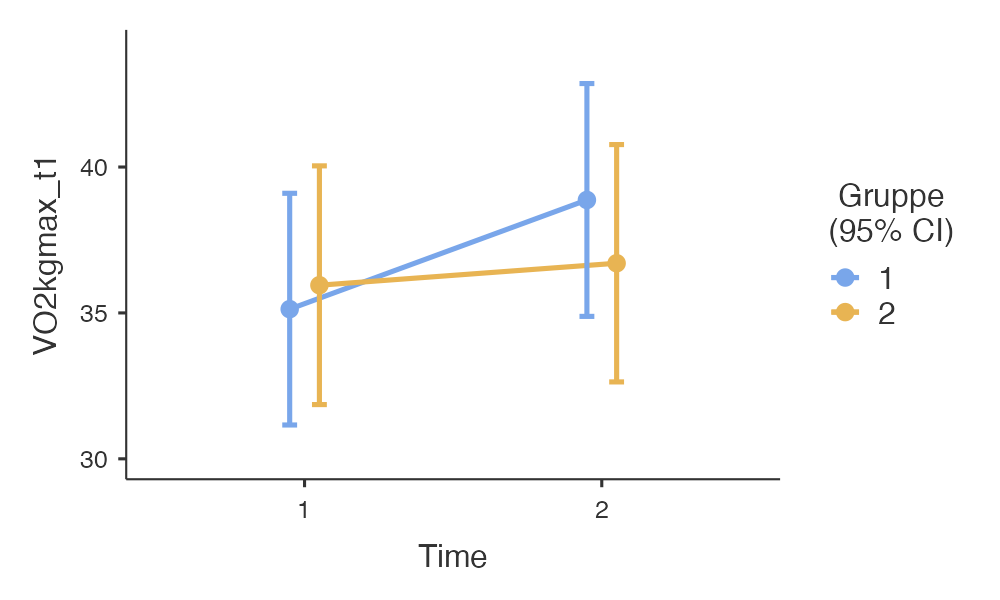


**Mixed Model MVPA**

| Model Info | | | |
| --- | --- | --- | --- |
| **Info** | |  | |
| Estimate |  | Linear mixed model fit by REML |  |
| Call |  | t1_MVPA ~ 1 + Gruppe + Time + Gewicht_t1kg + Gruppe:Time+( 1 \| ID ) |  |
| AIC |  | 1075.01 |  |
| BIC |  | 1056.14 |  |
| LogLikel. |  | -512.48 |  |
| R-squared Marginal |  | 0.01 |  |
| R-squared Conditional |  | 0.61 |  |
| Converged |  | yes |  |
| Optimizer |  | bobyqa |  |
|  | | | |

**Model Results**

| Fixed Effect Omnibus tests | | | | | | | | | |
| --- | --- | --- | --- | --- | --- | --- | --- | --- | --- |
|  | | **F** | | **Num df** | | **Den df** | | **p** | |
| Gruppe |  | 0.19 |  | 1 |  | 40.41 |  | 0.662 |  |
| Time |  | 0.23 |  | 1 |  | 41.13 |  | 0.635 |  |
| Gewicht_t1kg |  | 0.00 |  | 1 |  | 42.62 |  | 0.952 |  |
| Gruppe ✻ Time |  | 0.42 |  | 1 |  | 40.99 |  | 0.521 |  |
| Anmerkung. Satterthwaite method for degrees of freedom | | | | | | | | | |
|  | | | | | | | | | |

| Fixed Effects Parameter Estimates | | | | | | | | | | | | | | | | | |
| --- | --- | --- | --- | --- | --- | --- | --- | --- | --- | --- | --- | --- | --- | --- | --- | --- | --- |
|  | | | | | | | | **95% Confidence Interval** | | | |  | | | | | |
| **Names** | | **Effect** | | **Estimate** | | **SE** | | **Lower** | | **Upper** | | **df** | | **t** | | **p** | |
| (Intercept) |  | (Intercept) |  | 145.99 |  | 18.23 |  | 110.26 |  | 181.71 |  | 40.05 |  | 8.01 |  | < .001 |  |
| Gruppe1 |  | 2 - 1 |  | 17.34 |  | 39.43 |  | -59.94 |  | 94.61 |  | 40.41 |  | 0.44 |  | 0.662 |  |
| Time1 |  | 2 - 1 |  | 8.67 |  | 18.12 |  | -26.84 |  | 44.18 |  | 41.13 |  | 0.48 |  | 0.635 |  |
| Gewicht_t1kg |  | Gewicht_t1kg |  | 0.10 |  | 1.69 |  | -3.21 |  | 3.41 |  | 42.62 |  | 0.06 |  | 0.952 |  |
| Gruppe1 ✻ Time1 |  | 2 - 1 ✻ 2 - 1 |  | -23.43 |  | 36.20 |  | -94.38 |  | 47.52 |  | 40.99 |  | -0.65 |  | 0.521 |  |
|  | | | | | | | | | | | | | | | | | |

| Random Components | | | | | | | | | |
| --- | --- | --- | --- | --- | --- | --- | --- | --- | --- |
| **Groups** | | **Name** | | **SD** | | **Variance** | | **ICC** | |
| ID |  | (Intercept) |  | 103.71 |  | 10755.79 |  | 0.60 |  |
| Residual |  |  |  | 83.90 |  | 7039.84 |  |  |  |
| Anmerkung. Number of Obs: 86 , groups: ID 43 | | | | | | | | | |
|  | | | | | | | | | |

**Post Hoc Tests**

| Post Hoc Comparisons - Gruppe ✻ Time | | | | | | | | | | | | | | | | | | | |
| --- | --- | --- | --- | --- | --- | --- | --- | --- | --- | --- | --- | --- | --- | --- | --- | --- | --- | --- | --- |
| **Comparison** | | | | | | | | | |  | | | | | | | | | |
| **Gruppe** | | **Time** | |  | | **Gruppe** | | **Time** | | **Difference** | | **SE** | | **t** | | **df** | | **p_bonferroni_** | |
| 1 |  | 1 |  | - |  | 1 |  | 2 |  | -20.38 |  | 25.31 |  | -0.81 |  | 41.00 |  | 1.000 |  |
| 1 |  | 1 |  | - |  | 2 |  | 1 |  | -29.05 |  | 43.42 |  | -0.67 |  | 56.59 |  | 1.000 |  |
| 1 |  | 1 |  | - |  | 2 |  | 2 |  | -26.01 |  | 43.14 |  | -0.60 |  | 56.79 |  | 1.000 |  |
| 1 |  | 2 |  | - |  | 2 |  | 2 |  | -5.62 |  | 43.38 |  | -0.13 |  | 56.62 |  | 1.000 |  |
| 2 |  | 1 |  | - |  | 1 |  | 2 |  | 8.67 |  | 43.67 |  | 0.20 |  | 56.42 |  | 1.000 |  |
| 2 |  | 1 |  | - |  | 2 |  | 2 |  | 3.05 |  | 25.91 |  | 0.12 |  | 41.02 |  | 1.000 |  |
|  | | | | | | | | | | | | | | | | | | | |

**Estimated Marginal Means**

| Gruppe | | | | | | | | | | | |
| --- | --- | --- | --- | --- | --- | --- | --- | --- | --- | --- | --- |
|  | | | | | | | | **95% Confidence Interval** | | | |
| **Gruppe** | | **Mean** | | **SE** | | **df** | | **Lower** | | **Upper** | |
| 1 |  | 137.32 |  | 26.51 |  | 40.24 |  | 83.75 |  | 190.88 |  |
| 2 |  | 154.66 |  | 27.18 |  | 40.25 |  | 99.73 |  | 209.58 |  |
| Anmerkung. Estimated means are estimated averaging across interacting variables | | | | | | | | | | | |
|  | | | | | | | | | | | |

| Time | | | | | | | | | | | |
| --- | --- | --- | --- | --- | --- | --- | --- | --- | --- | --- | --- |
|  | | | | | | | | **95% Confidence Interval** | | | |
| **Time** | | **Mean** | | **SE** | | **df** | | **Lower** | | **Upper** | |
| 1 |  | 141.65 |  | 20.36 |  | 58.72 |  | 100.91 |  | 182.39 |  |
| 2 |  | 150.32 |  | 20.35 |  | 58.73 |  | 109.60 |  | 191.04 |  |
| Anmerkung. Estimated means are estimated averaging across interacting variables | | | | | | | | | | | |
|  | | | | | | | | | | | |

| Gruppe:Time | | | | | | | | | | | | | |
| --- | --- | --- | --- | --- | --- | --- | --- | --- | --- | --- | --- | --- | --- |
|  | | | | | | | | | | **95% Confidence Interval** | | | |
| **Gruppe** | | **Time** | | **Mean** | | **SE** | | **df** | | **Lower** | | **Upper** | |
| 1 |  | 1 |  | 127.13 |  | 29.29 |  | 57.74 |  | 68.50 |  | 185.76 |  |
| 2 |  | 1 |  | 156.18 |  | 30.22 |  | 57.48 |  | 95.68 |  | 216.67 |  |
| 1 |  | 2 |  | 147.51 |  | 29.46 |  | 57.55 |  | 88.53 |  | 206.49 |  |
| 2 |  | 2 |  | 153.13 |  | 30.01 |  | 57.71 |  | 93.06 |  | 213.20 |  |
| Anmerkung. Estimated means are estimated keeping constant other effects in the model to the mean | | | | | | | | | | | | | |
|  | | | | | | | | | | | | | |

**Effects Plots**


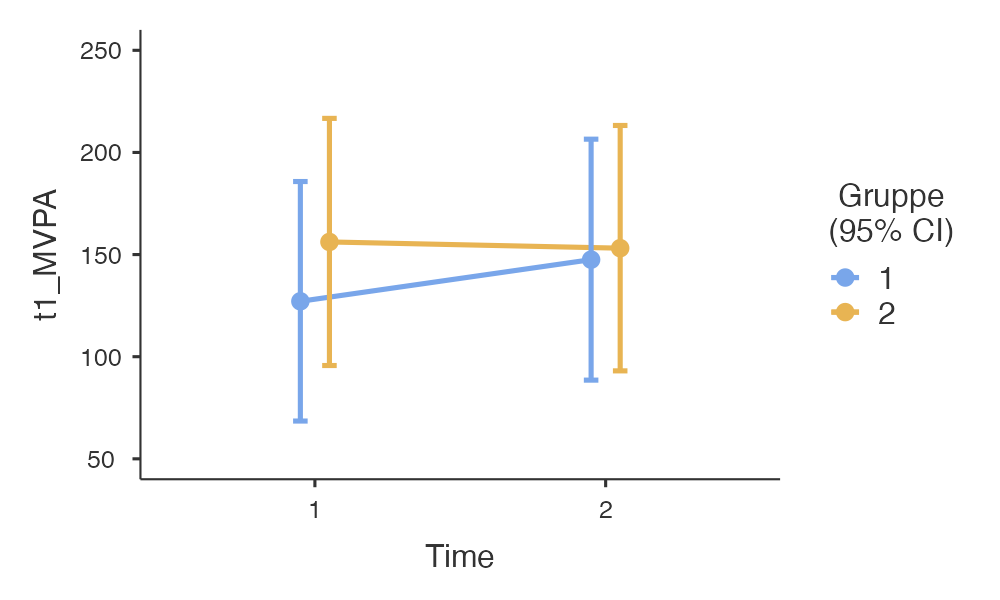


**Mixed Model PACES**

| Model Info | | | |
| --- | --- | --- | --- |
| **Info** | |  | |
| Estimate |  | Linear mixed model fit by REML |  |
| Call |  | PACES_t1_gesamt ~ 1 + Gruppe + Time + Gewicht_t1kg + Gruppe:Time+( 1 \| ID ) |  |
| AIC |  | 602.59 |  |
| BIC |  | 611.14 |  |
| LogLikel. |  | -289.98 |  |
| R-squared Marginal |  | 0.11 |  |
| R-squared Conditional |  | 0.68 |  |
| Converged |  | yes |  |
| Optimizer |  | bobyqa |  |
|  | | | |

**Model Results**

| Fixed Effect Omnibus tests | | | | | | | | | |
| --- | --- | --- | --- | --- | --- | --- | --- | --- | --- |
|  | | **F** | | **Num df** | | **Den df** | | **p** | |
| Gruppe |  | 5.41 |  | 1 |  | 40.48 |  | 0.025 |  |
| Time |  | 0.00 |  | 1 |  | 41.15 |  | 0.972 |  |
| Gewicht_t1kg |  | 0.00 |  | 1 |  | 42.97 |  | 0.957 |  |
| Gruppe ✻ Time |  | 0.01 |  | 1 |  | 41.00 |  | 0.909 |  |
| Anmerkung. Satterthwaite method for degrees of freedom | | | | | | | | | |
|  | | | | | | | | | |

| Fixed Effects Parameter Estimates | | | | | | | | | | | | | | | | | |
| --- | --- | --- | --- | --- | --- | --- | --- | --- | --- | --- | --- | --- | --- | --- | --- | --- | --- |
|  | | | | | | | | **95% Confidence Interval** | | | |  | | | | | |
| **Names** | | **Effect** | | **Estimate** | | **SE** | | **Lower** | | **Upper** | | **df** | | **t** | | **p** | |
| (Intercept) |  | (Intercept) |  | 65.86 |  | 1.21 |  | 63.50 |  | 68.22 |  | 40.07 |  | 54.62 |  | < .001 |  |
| Gruppe1 |  | 2 - 1 |  | -6.06 |  | 2.61 |  | -11.17 |  | -0.95 |  | 40.48 |  | -2.32 |  | 0.025 |  |
| Time1 |  | 2 - 1 |  | -0.04 |  | 1.13 |  | -2.25 |  | 2.17 |  | 41.15 |  | -0.04 |  | 0.972 |  |
| Gewicht_t1kg |  | Gewicht_t1kg |  | 0.01 |  | 0.11 |  | -0.21 |  | 0.22 |  | 42.97 |  | 0.05 |  | 0.957 |  |
| Gruppe1 ✻ Time1 |  | 2 - 1 ✻ 2 - 1 |  | -0.26 |  | 2.25 |  | -4.67 |  | 4.15 |  | 41.00 |  | -0.11 |  | 0.909 |  |
|  | | | | | | | | | | | | | | | | | |

| Random Components | | | | | | | | | |
| --- | --- | --- | --- | --- | --- | --- | --- | --- | --- |
| **Groups** | | **Name** | | **SD** | | **Variance** | | **ICC** | |
| ID |  | (Intercept) |  | 6.99 |  | 48.87 |  | 0.64 |  |
| Residual |  |  |  | 5.22 |  | 27.23 |  |  |  |
| Anmerkung. Number of Obs: 86 , groups: ID 43 | | | | | | | | | |
|  | | | | | | | | | |

**Post Hoc Tests**

| Post Hoc Comparisons - Gruppe ✻ Time | | | | | | | | | | | | | | | | | | | |
| --- | --- | --- | --- | --- | --- | --- | --- | --- | --- | --- | --- | --- | --- | --- | --- | --- | --- | --- | --- |
| **Comparison** | | | | | | | | | |  | | | | | | | | | |
| **Gruppe** | | **Time** | |  | | **Gruppe** | | **Time** | | **Difference** | | **SE** | | **t** | | **df** | | **p_bonferroni_** | |
| 1 |  | 1 |  | - |  | 1 |  | 2 |  | -0.09 |  | 1.57 |  | -0.06 |  | 41.00 |  | 1.000 |  |
| 1 |  | 1 |  | - |  | 2 |  | 1 |  | 5.93 |  | 2.84 |  | 2.09 |  | 54.89 |  | 0.249 |  |
| 1 |  | 1 |  | - |  | 2 |  | 2 |  | 6.10 |  | 2.82 |  | 2.16 |  | 55.06 |  | 0.210 |  |
| 1 |  | 2 |  | - |  | 2 |  | 2 |  | 6.19 |  | 2.84 |  | 2.18 |  | 54.92 |  | 0.201 |  |
| 2 |  | 1 |  | - |  | 1 |  | 2 |  | -6.02 |  | 2.86 |  | -2.11 |  | 54.74 |  | 0.239 |  |
| 2 |  | 1 |  | - |  | 2 |  | 2 |  | 0.17 |  | 1.61 |  | 0.10 |  | 41.02 |  | 1.000 |  |
|  | | | | | | | | | | | | | | | | | | | |

**Estimated Marginal Means**

| Gruppe | | | | | | | | | | | |
| --- | --- | --- | --- | --- | --- | --- | --- | --- | --- | --- | --- |
|  | | | | | | | | **95% Confidence Interval** | | | |
| **Gruppe** | | **Mean** | | **SE** | | **df** | | **Lower** | | **Upper** | |
| 1 |  | 68.89 |  | 1.75 |  | 40.28 |  | 65.35 |  | 72.44 |  |
| 2 |  | 62.83 |  | 1.80 |  | 40.29 |  | 59.20 |  | 66.46 |  |
| Anmerkung. Estimated means are estimated averaging across interacting variables | | | | | | | | | | | |
|  | | | | | | | | | | | |

| Time | | | | | | | | | | | |
| --- | --- | --- | --- | --- | --- | --- | --- | --- | --- | --- | --- |
|  | | | | | | | | **95% Confidence Interval** | | | |
| **Time** | | **Mean** | | **SE** | | **df** | | **Lower** | | **Upper** | |
| 1 |  | 65.88 |  | 1.33 |  | 56.76 |  | 63.21 |  | 68.55 |  |
| 2 |  | 65.84 |  | 1.33 |  | 56.77 |  | 63.18 |  | 68.51 |  |
| Anmerkung. Estimated means are estimated averaging across interacting variables | | | | | | | | | | | |
|  | | | | | | | | | | | |

| Gruppe:Time | | | | | | | | | | | | | |
| --- | --- | --- | --- | --- | --- | --- | --- | --- | --- | --- | --- | --- | --- |
|  | | | | | | | | | | **95% Confidence Interval** | | | |
| **Gruppe** | | **Time** | | **Mean** | | **SE** | | **df** | | **Lower** | | **Upper** | |
| 1 |  | 1 |  | 68.85 |  | 1.92 |  | 55.91 |  | 65.01 |  | 72.69 |  |
| 2 |  | 1 |  | 62.91 |  | 1.98 |  | 55.69 |  | 58.95 |  | 66.88 |  |
| 1 |  | 2 |  | 68.94 |  | 1.93 |  | 55.74 |  | 65.07 |  | 72.80 |  |
| 2 |  | 2 |  | 62.74 |  | 1.96 |  | 55.88 |  | 58.81 |  | 66.68 |  |
| Anmerkung. Estimated means are estimated keeping constant other effects in the model to the mean | | | | | | | | | | | | | |
|  | | | | | | | | | | | | | |

**Effects Plots**


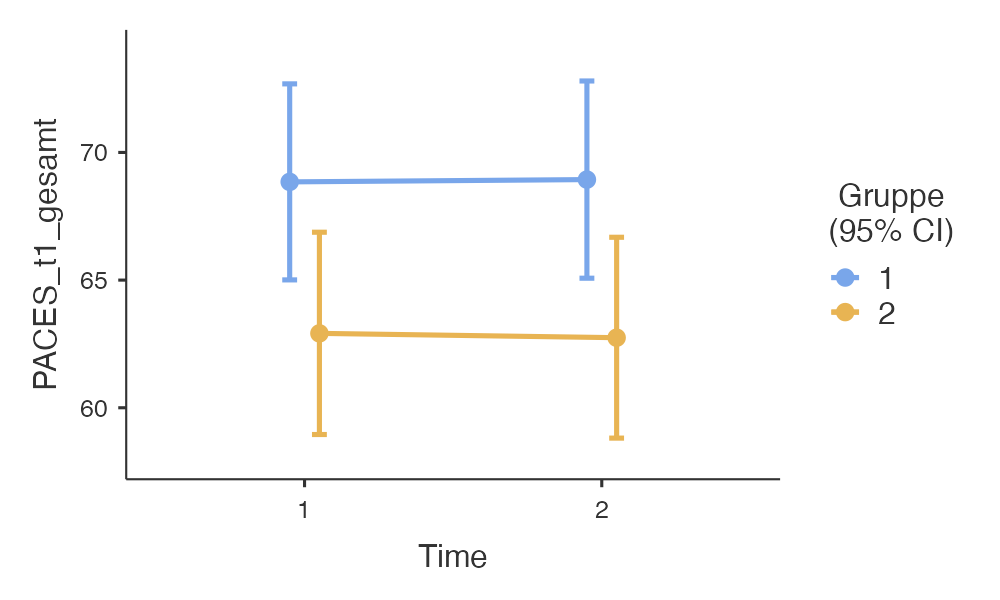

Supplement: Supplementary file 3 — Supplementary file3 (DOCX 670 KB) [file 421_2025_5743_MOESM3_ESM.docx]
